# Analysis of hospital resilience in public health emergencies using structural equation modeling: based on risk perception, resource preparedness, and team collaboration

**DOI:** 10.3389/fpubh.2026.1819684

**Published:** 2026-05-15

**Authors:** Yunpeng Chen, Hao Wu

**Affiliations:** 1 Peking Union Medical College Press, Beijing, China; 2 Department of Emergency, Beijing Jishuitan Hospital, Capital Medical University, Beijing, China

**Keywords:** hospital resilience, public health emergencies, resource governance, risk perception, structural equation modeling, team collaboration

## Abstract

**Background:**

Public health emergencies place sustained pressure on hospital operations and service continuity. However, existing studies have more often emphasized institutional arrangements or resource stockpiles than the governance processes through which hospital resilience is associated with risk sensing, resource orchestration, and collaborative operation.

**Objective:**

This study aimed to test a governance-based framework linking risk sensing, resource orchestration, collaborative governance, and hospital resilience in a public health emergency context.

**Methods:**

A retrospective observational design was used based on the COVID-19 response of our hospital from January to June 2020. Management archive data and retrospective questionnaire data were integrated, and 386 valid cases were analyzed using structural equation modeling with subgroup and robustness analyses.

**Results:**

The results showed that stronger risk sensing was positively associated with stronger resource orchestration and collaborative governance, and that collaborative governance had the strongest association with resilience. The direct association between risk sensing and resilience became non-significant after the mediating variables were included, supporting an indirect pathway through resource orchestration and collaboration. These patterns remained broadly stable across subgroup and robustness analyses.

**Conclusion:**

In this single-hospital COVID-19 context, hospital resilience was associated with a sequential governance pattern linking risk sensing, resource orchestration, and collaborative operation. The findings support a capability-oriented perspective on hospital emergency governance, while their applicability is most direct for large, relatively well-resourced hospitals.

## 1 Introduction

Public health emergencies, including disease outbreaks, climate-related disasters, conflict, and displacement, persistently challenge the continuity, safety, and adaptive capacity of healthcare systems (1). Hospitals, as critical operational nodes, must sustain clinical activity while absorbing shocks, adapting organizational processes, and restoring essential functions under high uncertainty (2). Although hospital resilience has become an important topic in recent health-system research, recent hospital-focused reviews and frameworks indicate that its conceptualization and measurement remain under development (2, 3). The conceptual vocabulary used in this study is grounded in post-2010 health-system resilience scholarship. In particular, Kruk et al. (4) conceptualize resilient health systems as those able to absorb disturbance and reorganize while maintaining core functions, while Blanchet et al. (5) further specify resilience in terms of absorptive, adaptive, and transformative capacities. Subsequent reviews and European policy work have extended this vocabulary to empirical health-system and hospital contexts (6, 7). However, key questions remain about how these resilience capacities are associated with concrete governance processes inside hospitals and how such processes can be conceptualized and measured.

From a governance perspective, hospital resilience requires more than just resource reserves or material redundancy. Research on hospital supply chain resilience indicates that resilience depends on dynamic capabilities—such as anticipation, adaptation, response, recovery, and learning—rather than on static stockpiles alone (8). This study draws on dynamic capability theory, which views organizational adaptation as the integration and reconfiguration of internal and external competences in changing environments. We link hospital resilience to a chain of organizational processes: risk sensing, resource orchestration, and collaborative execution (9). This framework is also informed by Teece’s sensing–seizing–reconfiguring microfoundations (10) and by Eisenhardt and Martin’s (11) view of dynamic capabilities as organizational processes that enable resource recombination under uncertainty.

Existing studies show that disaster risk perception among hospital personnel varies by occupational role and professional background. This perception may shape how staff prepare for emergencies (12). However, reviews of risk perception during public health emergencies have largely focused on individual, contextual, and media-related factors. As a result, there is limited structural evidence on how organizational risk sensing translates into downstream governance capabilities and, ultimately, into hospital resilience (13).

A second gap concerns emergency resource preparedness and governance capability. Prior studies on hospital supply chain resilience, surge capacity planning, and crisis resource management suggest that hospital performance under shock depends less on how many resources are stocked than on how those resources are anticipated, allocated, embedded in operations, and reconfigured under pressure (8, 14). Yet many studies still treat resource preparedness as a static capacity or stock condition rather than as an orchestration capability. This makes it difficult to explain why hospitals with similar resource bases perform differently under comparable stress (8, 14).

A third gap concerns collaborative governance. In high-pressure emergencies, hospital resilience depends not only on resources but also on command clarity, information flow, team leadership, and cross-departmental coordination (15, 16). However, collaboration is still often treated as a contextual condition rather than as a measurable governance capability in resilience models (16). Moreover, how collaboration interacts with risk sensing and resource orchestration within a single structural framework remains underexamined (16).

A further limitation is the geographic and institutional concentration of the literature. European research on health-system resilience emphasizes that resilience should be understood across the full shock cycle, including preparedness, shock management, recovery, and learning (7). In hospital care specifically, cross-national evidence from five countries shows that pre-existing care structures and capacity-expansion strategies shaped how hospitals coped with the first wave of COVID-19 (17). Together with broader empirical reviews of health-system resilience (18), these studies suggest that findings from a single large tertiary hospital are institutionally bounded and not universally generalizable.

These gaps are important because hospitals increasingly face compound and cross-domain risks, including infectious disease outbreaks and disaster-related disruptions. Reviews of healthcare organizational resilience support the need to study resilience across complex operational settings (16). Recent health-system resilience frameworks emphasize absorptive, adaptive, and transformative capacities as core concepts (5). European policy work situates resilience across preparedness, shock management, recovery, and learning phases (7). Empirical reviews further show that resilience research has moved toward system-level and multi-dimensional explanations (6, 18). More recent frameworks extend this logic to national, regional, local, and organizational levels (19). Taken together, this literature supports assessing hospital performance through governance processes and multi-dimensional indicators, rather than through single-event or single-resource perspectives alone.

Against this background, the present study addresses the following question: how are risk sensing, resource orchestration, and collaborative governance jointly associated with hospital resilience during a public health emergency? The study makes three contributions. First, it tests a serial mediation framework rather than examining only direct associations among the main constructs. Second, it conceptualizes hospital resilience from a governance-capability perspective rather than treating it solely as an outcome variable. Third, it integrates risk sensing, resource orchestration, and collaborative governance into a single structural framework, thereby extending prior research that often addressed these dimensions separately. To examine these issues, the study uses retrospective multi-source data and structural equation modeling in the context of a large tertiary hospital’s COVID-19 response.

## 2 Methods

### 2.1 Study design and analysis

This retrospective observational study used our hospital’s operational process during the initial COVID-19 response (January–June 2020) to examine how risk perception, resource preparedness, and team collaboration influence hospital resilience. We analyzed hospital organizational units as risk governance subjects embedded in the public health emergency response system, focusing on risk identification, capability configuration, and collaborative execution under high uncertainty.

Based on emergency risk management logic and organizational resilience theory, we defined hospital resilience as the ability to maintain functions, recover rapidly, reconfigure resources, and learn from sudden shocks. We constructed an analytical framework: “risk identification → capability configuration → collaborative execution → system reconfiguration.” In this framework, risk perception represents organizational risk identification; resource preparedness reflects the emergency capability foundation; team collaboration characterizes cross-departmental governance quality; hospital resilience is the outcome variable.

We employed a retrospective multi-source data integration scheme, combining management archive and survey data to reduce single-source bias and improve measurement validity. Given the infeasibility of experimental interventions in emergencies, we identified association patterns through structured retrospective review. Structural equation modeling (SEM) estimated direct and indirect effects among latent variables simultaneously, capturing coexisting pathways and nested relationships.

### 2.2 Research context, time window, and institutional background

During the study period, the hospital had approximately 1,500 licensed beds, over 40 clinical departments, and about 3,200 employees. Core emergency-related units included the emergency department, ICU, fever clinic/isolation units, infection control, respiratory care, laboratory medicine, imaging, logistics, and administration. As a typical large public hospital, it performed regional treatment, resource coordination, emergency referral, and information reporting within national and local public health emergency response systems. Its emergency response relied on standing command and coordination mechanisms, achieving resource allocation and process reconfiguration through cross-departmental linkage. During the initial COVID-19 outbreak, the hospital maintained a dedicated fever clinic, isolation and triage procedures, infection-control protocols, reserve management for PPE and critical supplies, and a cross-departmental emergency command structure. These features indicate mature emergency and infectious-disease response capacity, providing a comparable institutional background. This study focuses on the initial COVID-19 outbreak—a context of extraordinary policy mobilization and institutional pressure—which should be considered when interpreting results.

The study window (January–June 2020) covered early warning, emergency response, and phased recovery in Beijing. To enhance procedural consistency, we divided the window into three phases based on process characteristics and operational load: Phase 1 (early warning and preparation, T1): risk information accumulation, system activation, resource prepositioning. Phase 2 (shock and high-load period, T2): increased medical demand, concentrated organizational pressure, intensive cross-departmental collaboration. Phase 3 (recovery and reconfiguration, T3): process reconfiguration, resource adjustment, institutional improvements. This delineation defines context boundaries and retrospective measurement anchors. Because the study occurred during the initial COVID-19 outbreak, observed governance patterns may partly reflect emergency state mobilization; generalization to routine conditions or other emergencies requires caution.

We defined core constructs around this temporal structure. Risk perception corresponds to T1–T2 risk identification; resource preparedness to T1 capability configuration; team collaboration to T2 collaborative governance quality; hospital resilience to T2–T3 functional maintenance and recovery outcomes. This time-windowing preserves phased characteristics of emergency governance within a retrospective design, improving contextual relevance. As a large tertiary Grade A public hospital in Beijing with mature emergency command capacity, the study hospital is informative for highly resourced settings but not representative of lower-tier or resource-constrained hospitals.

### 2.3 Study participants, sampling strategy, and sample structure

#### 2.3.1 Study participants

Participants were on-duty staff who participated in or supported the hospital’s COVID-19 response during January–June 2020, covering medical treatment, infection control, resource support, and organizational coordination. The retrospective questionnaire asked respondents to evaluate their experiences during this period. Participants included clinical physicians, nursing staff, medical technicians, infection-control personnel, logistics support staff, and administrative staff. The final sample included 110 clinical physicians (28.5%), 122 nursing staff (31.6%), 57 medical technicians (14.8%), 29 infection-control/public health staff (7.5%), 37 logistics and support personnel (9.6%), and 31 administrative and management staff (8.0%), covering major emergency-response functions.

#### 2.3.2 Inclusion and exclusion criteria

Inclusion criteria: (1) Actual participation in COVID-19 response tasks (medical treatment, infection control, resource support, or organizational coordination) during January–June 2020. (2) Continuous employment ≥6 months with stable positions before and after the study window. (3) Normal on-duty status during the window (no long-term sick leave, off-duty training, or external assignments). (4) Access to the study information statement and voluntary participation. (5) Ability to complete all questionnaire items independently.

Exclusion criteria: (1) Participation only through short-term rotation, temporary support, secondment, or external aid. (2) No substantive engagement in emergency response or only peripheral tasks. (3) Frequent job changes or major responsibility adjustments during the study window. (4) Questionnaires with severe missing data, repetitive response patterns, extreme homogeneous responses, or inconsistencies in reverse-scored or logic-related items. (5) Response times too short or abnormally concentrated. (6) Explicit indication of lacking basic understanding of emergency processes.

#### 2.3.3 Sampling strategy and sample size

We used stratified convenience sampling. Without compromising anonymity and voluntariness, we structured coverage by job type (clinical, nursing, medical technology, infection control, support management) and department (high-coupling units: emergency, ICU, fever clinic, infection control, logistics; and management departments). Department liaisons disseminated survey information and reminded participants but did not access individual responses, reducing selection pressure and social desirability bias.

Sample size justification was based on SEM parameter estimation stability, considering measurement model item scale, number of latent variables, and potential design effects. Target sample size accounted for (1) the ratio of observed indicators to free parameters, (2) fit index sensitivity to sample size, and (3) variance inflation from stratified sampling. The resulting sample size met statistical power requirements. Detailed sample size, collection, and exclusion processes are documented in Supplementary Appendix Section S6.

### 2.4 Data sources and multi-source integration procedures

We used a process-based multi-source data integration strategy, involving unified collection, cleaning, matching, and modeling of data from different sources. All data collection and integration were completed by the research team and hospital information management department following unified specifications and quality control procedures.

#### 2.4.1 Management archives and operational data

Archive and operational data came from the Hospital Information System (HIS), emergency command platform, human resource system, and material management system. The hospital information management department extracted data following our variable definitions, covering operational records directly related to emergency response within the study window.

Extraction and processing steps: (1) Export raw data tables with complete timestamps, department codes, and job identifiers. (2) Verify field consistency and completeness, cross-referencing original business logs and management documents. (3) De-identify verified data to form a structured research dataset.

Archive data included emergency material reserve and allocation records, training archives, manpower dispatch logs, cross-departmental meeting minutes and bulletins, and key performance indicators reflecting service continuity and operational load. For unstructured text (meeting minutes, bulletins), two researchers independently coded key event nodes and institutional adjustments, with consistency checks. All archive data were converted into aggregated indicator forms by study phase before analysis and jointly reviewed for temporal consistency and statistical caliber stability.

#### 2.4.2 Retrospective survey data

We collected retrospective survey data via a structured electronic questionnaire. All items explicitly referred to respondents’ experiences during the January–June 2020 COVID-19 response. The questionnaire was distributed centrally 3–6 months after the event using a unified platform link pushed to eligible staff, with a study information statement released through the hospital information system before distribution.

To reduce recall bias, the questionnaire homepage included a standardized timeline and prompts for key institutional milestones (emergency command activation, infection-control escalation, resource coordination phases) as reference anchors. Each item clearly specified the evaluation object and time range.

The survey platform automatically recorded response times, modification trails, and interruption status. We screened raw data for abnormal response durations, repetitive patterns, and inconsistencies in key reverse-scored items. Data passing screening entered cleaning and analysis. Because the questionnaire relied on retrospective self-report, recall bias cannot be fully excluded, although standardized timelines and key-event prompts were used to mitigate this risk.

#### 2.4.3 Data integration and quality control procedures

We followed a standardized technical pathway for data integration and quality control:

(1) Cross-system matching and anonymous linking: With hospital information management assistance, we generated anonymous unique identification codes for all participants, enabling linkage between questionnaire and archive data without personal identifiers.
(2) Cross-source consistency verification: For key variables (resource preparedness, collaboration frequency, workload), we checked trend consistency and distribution rationality between subjective survey results and objective operational records. Observations with significant deviations were reviewed against original records.
(3) Variable structuring and standardization: Data passing verification underwent structural transformation and unit harmonization. Continuous variables were range- or Z-score standardized. Categorical variables were dummy-coded. Time-series indicators were aggregated by study phase.
(4) Missing and anomalous data correction: We analyzed missing patterns and detected outliers. For variables with low random missingness, we used multiple imputation or robust estimation comparatively. For extreme outliers, we assessed impact via sensitivity analysis and trimmed or removed as necessary.
(5) Structural embedding for integrated modeling: Subjective survey data were used to estimate the latent variable measurement model. Management archive data were embedded as external reference indicators and robustness variables, estimated jointly through covariance paths or alternative regression models.
(6) Process documentation and cross-verification: We maintained a systematic data processing log detailing all cleaning, matching, transformation, and correction operations, ensuring traceability and reproducibility.

Through these phased, auditable procedures, we maximized retention of structural information from the emergency risk governance process within a retrospective design, providing a reliable data foundation for SEM analysis.

### 2.5 Variable measurement and scale development

Drawing on Teece’s dynamic capability framework, we conceptualized risk perception, resource preparedness, and team collaboration as capability-oriented processes related to sensing, resource orchestration, and coordinated execution (9, 10). Eisenhardt and Martin’s (11) process-based view further supported treating these capabilities as organizational routines rather than static attributes. Hospital resilience was defined using the absorptive, adaptive, and transformative capacity vocabulary established in post-2010 health-system resilience scholarship (4, 5). European policy and empirical resilience reviews informed the contextual interpretation of this definition (19, 20). The measurement model and SEM procedures followed Kline’s structural equation modeling guidance (20). The full bilingual disclosure version of questionnaire items, item sources, and response anchors is provided in Supplementary Appendix Table S1.

#### 2.5.1 Scale sources and contextualization procedures

(1) Construct tracing and literature mapping: We systematically searched Web of Science, Scopus, and PubMed for literature on risk perception, emergency resource management, collaborative governance, and organizational resilience, compared conceptual definitions, dimensional structures, and measurement approaches, and built a preliminary conceptual mapping framework.
(2) Translation and back-translation: For items from foreign-language literature, we followed bidirectional translation and independent back-translation by bilingual researchers with management and medical backgrounds, with multiple rounds of expert discussion to correct semantic deviations.
(3) Contextual item reconceptualization: While maintaining original theoretical dimensions, we rewrote preliminary items to incorporate the hospital’s emergency command system, resource allocation processes, and interdepartmental collaboration mechanisms, ensuring items referred to specific management behaviors and institutional processes.
(4) Expert review and iterative revision: Multiple experts in emergency management, hospital management, and organizational behavior reviewed items for theoretical consistency, institutional compatibility, and operational comprehensibility. Items were revised based on feedback. The final bilingual item set is in Supplementary Appendix Table S1; construct definitions are in Supplementary Appendix Table S2.

#### 2.5.2 Content validity assessment and pilot testing

(1) Quantitative content validity assessment: Domain experts independently rated each item’s relevance, representativeness, and clarity. Items below recommended thresholds (CVI or Aiken’s V) were revised or removed.
(2) Pilot testing and cognitive consistency check: A small-scale sample with cognitive interviews assessed consistency in item understanding, temporal references, and contextual orientation. Items were optimized based on ambiguous interpretations and response difficulties.
(3) Preliminary reliability screening: Based on pilot test data, we calculated internal consistency coefficients for each latent variable and corrected or deleted items that significantly reduced reliability.

#### 2.5.3 Capability-oriented measurement design for core latent variables

All items used a 5-point Likert scale (1 = Strongly Disagree, 5 = Strongly Agree), with higher scores indicating higher governance capability. Full item wording is in Supplementary Appendix Table S1.

(1) Risk sensing and interpretation capability: Ability to identify, interpret, and provide early warning responses to risk information. Dimensions: Information Sensing Density, Risk Interpretation Consistency, Warning Response Speed, Uncertainty Handling Capability.
(2) Emergency resource orchestration capability: Ability to coordinate, configure, allocate, and dynamically reconfigure critical resources. Dimensions: Resource Deployability, Strategic Redundancy Configuration, Resource Reconfiguration Capability, Decision Embeddedness.
(3) Collaborative governance network capability: Collaborative operational quality of the multi-department emergency governance network. Dimensions: Command Network Clarity, Information Flow Efficiency, Interdepartmental Trust Capital, Collaborative Adaptability.
(4) Dynamic governance resilience: Ability to absorb risk, reconfigure functions, and engage in institutional learning. Dimensions: Shock Absorption Capacity, Functional Reconfiguration Capacity, Institutional Learning Capacity, Risk Memory Capacity.

#### 2.5.4 Cross-measurement design with semi-objective indicators

To reduce common source bias, we incorporated operational indicators from management archives as semi-objective reference variables: risk warning initiation delay time, key material allocation approval duration, cross-departmental coordination meeting frequency, and core service recovery cycle. These were used for trend comparison and consistency checks, not directly in main structural model estimation.

#### 2.5.5 Control variables

Based on existing research, we selected individual and organizational characteristics that might influence risk cognition, resource allocation, and performance outcomes: gender (0 = male, 1 = female), age (1 = <25, 2 = 25–34, 3 = 35–44, 4 = ≥45), education (1 = associate or below, 2 = bachelor, 3 = master or above), work tenure (1 = <5 years, 2 = 5–10, 3 = 11–20, 4= > 20), job type (dummy-coded, frontline clinical/nursing as reference), department attributes (0 = low-risk, 1 = high-risk: emergency, ICU, fever clinic), frontline care participation (0 = no, 1 = yes), and management level (0 = general staff, 1 = middle/senior management). These were entered as covariates in the structural model. Full coding is in Supplementary Appendix Table S3.

### 2.6 Data preprocessing and statistical assumption testing

We performed systematic data preprocessing before measurement and structural model estimation.

(1) Missing value analysis: Analyzed missing proportion, distribution, and mechanism (MCAR, MAR, MNAR) using Little’s MCAR test. For acceptable missing proportion, used multiple imputation and EM algorithms comparatively, comparing key statistics before and after imputation.
(2) Multivariate outlier identification: Calculated Mahalanobis distances against chi-square critical values. For identified outliers, conducted case diagnostics and sensitivity analysis, deciding retention, trimming, or removal under uniform standards.
(3) Normality testing: Tested univariate normality using skewness and kurtosis and multivariate normality using Mardia’s coefficient. For deviations, recorded degree and employed robust estimation methods (MLR, Satorra–Bentler correction, or Bootstrap SE correction).
(4) Multicollinearity diagnosis: Calculated VIF and tolerance indicators. For VIF above thresholds, examined correlation matrices and theoretical logic to determine interference with structural path estimation.
(5) Response pattern screening: Screened for extreme response tendencies, straight-lining, low-variance responses, and excessive consistency using intra-individual variance, response entropy, and response time distribution. Anomalous samples were reviewed and decisions made according to preset standards.
(6) Preparation for robust estimation: Based on comprehensive assessment, pre-specified robust estimation strategies for data with significant distributional deviations.

### 2.7 Measurement model and structural model estimation strategy

We followed the “measurement-first” principle: first testing the measurement model’s reliability and validity, then proceeding to structural path estimation.

#### 2.7.1 Exploratory and confirmatory factor analysis

(1) Exploratory factor analysis: Performed EFA on pilot test samples and randomly split sub-samples using KMO and Bartlett’s test, principal axis factoring with Promax oblique rotation, and parallel analysis with scree plot to determine factor retention.

(2) Confirmatory factor analysis: After clarifying latent structure, conducted CFA on independent samples to estimate factor loadings, error terms, and latent variable correlations. Calculated CR and AVE for convergent validity and tested discriminant validity using Fornell–Larcker criterion and HTMT ratio.

(3) Measurement invariance and model modification: Where needed, tested measurement invariance across key subgroups (e.g., job type, frontline participation), assessing configural, metric, and scalar invariance. Model modifications made only with theoretical justification.

#### 2.7.2 Structural equation model estimation and path analysis

(1) Structural model construction: After measurement model validation, built a structural model incorporating risk perception, resource preparedness, team collaboration, and hospital resilience. Estimated direct and indirect effects simultaneously using robust maximum likelihood or weighted least squares based on data distribution.
(2) Model fit assessment: Assessed fit using *χ*^2^/df, CFI, TLI, RMSEA, and SRMR against recommended thresholds. Also built alternative models (removing direct paths, adjusting mediation order) for fit comparison.
(3) Path stability and sensitivity analysis: Re-estimated models using different estimation methods and sample subsets to compare changes in path coefficients.

#### 2.7.3 Mediation and chain mediation effect testing

(1) Indirect effect testing: Used non-parametric Bootstrap with 5,000 resamples to estimate indirect effects, calculating bias-corrected 95% confidence intervals.
(2) Chain mediation path modeling: Built a chain mediation model (risk perception → resource preparedness → team collaboration → hospital resilience) to estimate multiple transmission pathways simultaneously.
(3) Mediation effect robustness verification: Verified mediation stability using alternative model estimation and subgroup analysis.

### 2.8 Endogeneity control and robustness testing

We implemented multi-level robustness tests and bias control strategies to enhance causal interpretation credibility.

#### 2.8.1 Common method bias control

(1) Procedural controls: Used anonymous surveying, multi-source data integration, random item ordering, temporal anchoring, interleaved independent and dependent variable items, and avoided causal phrasing.
(2) Statistical testing: Harman’s single-factor test showed the first unrotated factor accounted for 31.8% of variance (below 40%). Full-collinearity diagnostics showed VIF values for core latent constructs ranged 1.82–2.64 (below 3.3). Adding an unmeasured latent method factor yielded minimal fit improvement (ΔCFI = 0.005, ΔRMSEA = −0.005) and limited path coefficient changes (Δ*β* = 0.02–0.05), indicating common method bias did not materially alter substantive conclusions.

#### 2.8.2 Alternative model comparison and structural stability testing

We constructed alternative structural models: removing the direct path from risk perception to resilience, reversing mediation order, and using parallel mediation. Compared fit indices, AIC, and BIC across models.

#### 2.8.3 Subgroup analysis and structural heterogeneity testing

Divided the sample by job type, frontline care participation, department attributes, and management level. Before multi-group comparisons, tested measurement invariance across groups. Then estimated structural models separately for each subgroup and tested path coefficient differences.

#### 2.8.4 Objective indicator comparison and external consistency validation

Selected operational indicators from management archives (risk warning delay, material approval duration, coordination frequency, service recovery cycle) as external reference variables. Built comparative regression models and compared results with the main SEM model.

### 2.9 Ethical compliance and data governance

The study protocol was approved by the Ethics Committee of Beijing Jishuitan Hospital (Approval No. 2020-K-042-01) before data collection. For the questionnaire component, respondents received a study information statement on the survey homepage, including research purpose, voluntary participation, and right to withdraw. Participants were required to read and check an electronic consent box before proceeding.

We implemented graded de-identification and anonymization for both questionnaire and archive data. All personally identifiable information was removed or replaced with codes before analysis. Only anonymous linkage codes were retained for cross-source matching. We followed the data minimization principle.

All research data were stored on encrypted servers with restricted access through hierarchical permission management. Only core research team members were authorized to access relevant data. Access, modification, and export operations were automatically logged and regularly audited.

After study completion, raw and processed data were archived according to institutional regulations. Data involving personal information will be securely destroyed or irreversibly anonymized after the legally required retention period. Any data sharing or secondary use requires re-approval from the Ethics Committee. Additional ethics and data-governance details are in Supplementary Appendix Section S7.

### 2.10 Software and computing platform

Data management and statistical analysis were completed in a standardized computing environment using a multi-software collaborative workflow to enhance result reproducibility and robustness. Data organization, variable coding, descriptive statistical analysis, and preliminary correlation testing were primarily conducted in IBM SPSS Statistics 26.0 (IBM Corp., Armonk, NY, United States), serving as data preparation for subsequent structural modeling by completing data structure checks, distribution feature summaries, and correlation matrix generation.

SEM analysis was primarily performed in AMOS 24.0 (IBM SPSS AMOS, IBM Corp., Armonk, NY, United States), Mplus 8.3 (Muthén & Muthén, Los Angeles, CA, United States), and R 4.2.2 (R Foundation for Statistical Computing, Vienna, Austria). Within the R platform, the lavaan 0.6–15 package was mainly used for CFA, structural model estimation, and mediation effect testing. During model estimation, appropriate estimators and robust correction methods were selected based on data distribution characteristics. Key model results were cross-validated across different software platforms, with particular focus on comparing consistency of fit indices, path coefficient directions, and significance levels, to minimize potential impact of software implementation differences on research conclusions.

Robustness testing and sensitivity analysis were primarily conducted in the R environment, utilizing the mice 3.14.0 package for multiple imputation analysis, the boot 1.3–28 package for Bootstrap resampling estimation, and the semTools 0.5–6 package for extended reliability/validity testing and model robustness assessment. All analysis scripts, parameter settings, and version information were systematically archived electronically, ensuring that the entire process—from data preprocessing to model estimation—possesses a foundation for repeatable execution and technical audit.

## 3 Results

### 3.1 Sample characteristics and preliminary bivariate analyses

**Figure 1:**
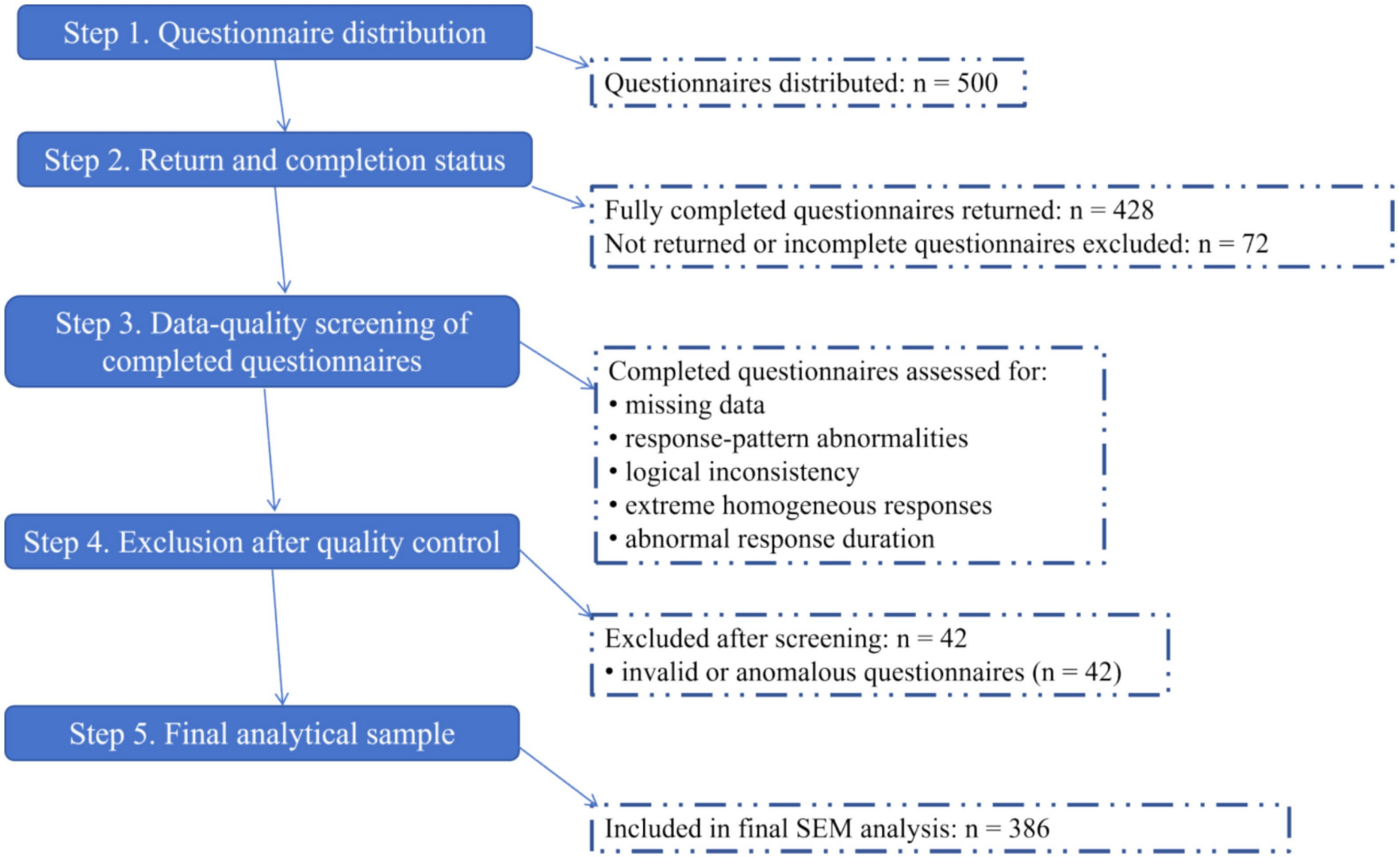
Flowchart of questionnaire distribution, screening, exclusion, and final sample inclusion. Of 500 distributed questionnaires, 428 were fully completed and 42 were excluded after quality-control screening, resulting in a final analytical sample of 386.

Figure 1 presents the participant screening process. Of 500 questionnaires distributed, 428 were fully completed. After missing value, response pattern, and logical consistency checks, 42 were excluded as invalid, leaving a final sample of 386 (effective response rate 90.2%). Sample quality met SEM requirements. A stepwise summary is in Supplementary Appendix Section S6.

Table 1 shows occupational composition: clinical physicians 28.5% (*n* = 110), nursing staff 31.6% (*n* = 122), medical technicians 14.8% (*n* = 57), infection control staff 7.5% (*n* = 29), logistics personnel 9.6% (*n* = 37), administrative staff 8.0% (*n* = 31). Frontline personnel accounted for 56.7% (*n* = 219), management and support roles 43.3% (*n* = 167).

Demographic characteristics (Table 1): Males 46.1% (*n* = 178), females 53.9% (*n* = 208). Age concentrated in 25–45 years (71.5%): 25–34 years 34.7%, 35–44 years 36.8%, ≥45 years 20.2%, <25 years 8.3%. Education: bachelor’s 52.3%, master’s or higher 27.5%, associate or below 20.2%. Work experience: <5 years 21.0%, 5–10 years 29.3%, 11–20 years 34.5%, >20 years 15.2%.

Descriptive statistics (Table 2): All latent variable scores in medium-to-high range. Risk sensing mean 3.82 (SD = 0.64), resource orchestration mean 3.76 (SD = 0.59), collaborative governance mean 3.69 (SD = 0.62), dynamic resilience mean 3.74 (SD = 0.61). SDs ranged 0.59–0.64, indicating adequate variance.

Pearson correlations (Table 2): Risk sensing with resource orchestration (*r* = 0.58, *p* < 0.001), with collaborative governance (*r* = 0.51, *p* < 0.001), with dynamic resilience (*r* = 0.43, *p* < 0.001). Resource orchestration with collaborative governance (*r* = 0.62, *p* < 0.001), with dynamic resilience (*r* = 0.57, *p* < 0.001). Collaborative governance with dynamic resilience (*r* = 0.60, *p* < 0.001). Correlations (0.43–0.62) were below multicollinearity thresholds, supporting discriminant validity.

**Table 1:** Institutional background, sample characteristics, and group differences (*N* = 386).

Baseline indicator	Total sample *n* (%)/mean ± SD	Frontline (*n* = 219)	Non-frontline (*n* = 167)	*χ*^2^/*t*	*p*-value
Institutional background					
Licensed beds	1,500	—	—	—	—
Number of clinical and medical-technical departments	42	—	—	—	—
Total workforce	3,200	—	—	—	—
Emergency-related core units established during study period	Fever clinic, ICU, emergency department, isolation/triage units, infection control, laboratory medicine, imaging, logistics support, hospital administration	—	—	—	—
Occupational composition of the sample					
Clinical physicians	110 (28.5)	110 (50.2)	0 (0.0)	—	—
Nursing staff	122 (31.6)	109 (49.8)	13 (7.8)	—	—
Medical technicians	57 (14.8)	0 (0.0)	57 (34.1)	—	—
Infection control/public health-related staff	29 (7.5)	0 (0.0)	29 (17.4)	—	—
Logistics and support personnel	37 (9.6)	0 (0.0)	37 (22.2)	—	—
Administrative and management staff	31 (8.0)	0 (0.0)	31 (18.6)	—	—
Demographic and work-related characteristics					
Gender				0.856	0.355
Male	178 (46.1)	96 (43.8)	82 (49.1)		
Female	208 (53.9)	123 (56.2)	85 (50.9)		
Age (years)	37.1 ± 9.2	34.8 ± 8.2	40.2 ± 9.6	−5.83	<0.001
Marital status				6.79	0.034
Unmarried	118 (30.6)	79 (36.1)	39 (23.4)		
Married/cohabiting	254 (65.8)	134 (61.2)	120 (71.9)		
Other	14 (3.6)	6 (2.7)	8 (4.8)		
Dependent family members				9.52	0.002
Yes	214 (55.4)	107 (48.9)	107 (64.1)		
No	172 (44.6)	112 (51.1)	60 (35.9)		
Education level				3.2	0.202
Junior college or below	78 (20.2)	38 (17.4)	40 (24.0)		
Bachelor	202 (52.3)	122 (55.7)	80 (47.9)		
Master or above	106 (27.5)	59 (26.9)	47 (28.1)		
Professional title				12.41	0.002
Junior	146 (37.8)	97 (44.3)	49 (29.3)		
Intermediate	173 (44.8)	94 (42.9)	79 (47.3)		
Senior	67 (17.4)	28 (12.8)	39 (23.4)		
Years of experience (years)	11.1 ± 7.6	9.1 ± 6.4	13.8 ± 8.1	−6.17	<0.001
Employment type				1.94	0.164
Permanent/long-term	284 (73.6)	155 (70.8)	129 (77.2)		
Temporary	102 (26.4)	64 (29.2)	38 (22.8)		
Management level				18.57	<0.001
None	296 (76.7)	186 (84.9)	110 (65.9)		
Supervisor	58 (15.0)	25 (11.4)	33 (19.8)		
Middle/senior	32 (8.3)	8 (3.7)	24 (14.4)		
Department risk level				49.26	<0.001
High-risk units	154 (39.9)	121 (55.3)	33 (19.8)		
Other units	232 (60.1)	98 (44.7)	134 (80.2)		
Shift work				28.31	<0.001
Yes	236 (61.1)	159 (72.6)	77 (46.1)		
No	150 (38.9)	60 (27.4)	90 (53.9)		
Weekly working hours	48.6 ± 10.4	52.1 ± 10.6	44.1 ± 8.2	8.32	<0.001
Night shifts per month	5.1 ± 4.0	6.3 ± 4.2	3.6 ± 3.1	7.05	<0.001
Emergency training (12 months)				22.89	<0.001
Yes	262 (67.9)	168 (76.7)	94 (56.3)		
No	124 (32.1)	51 (23.3)	73 (43.7)		
Participation in command system				45.44	<0.001
Yes	132 (34.2)	104 (47.5)	28 (16.8)		
No	254 (65.8)	115 (52.5)	139 (83.2)		
Certified in emergency management				5.44	0.02
Yes	88 (22.8)	58 (26.5)	30 (18.0)		
No	298 (77.2)	161 (73.5)	137 (82.0)		

Institutional background variables are reported at the hospital level and were therefore not subjected to frontline versus non-frontline group comparison. Frontline staff mainly included clinical physicians and nursing staff directly involved in emergency care and response; non-frontline staff included medical technicians, infection-control/public health staff, logistics/support personnel, and administrative/management staff.

**Table 2:** Extended descriptive statistics and correlation matrix (*N* = 386).

Variable	Mean	SD	1	2	3	4	5	6	7	8	9
1. Risk information sensitivity	3.85	0.62	1								
2. Risk interpretation consistency	3.78	0.65	0.61***	1							
3. Uncertainty handling capability	3.74	0.63	0.54***	0.59***	1						
4. Resource flexibility	3.79	0.58	0.49***	0.52***	0.55***	1					
5. Resource redundancy	3.68	0.61	0.44***	0.47***	0.50***	0.62***	1				
6. Decision embeddedness	3.73	0.6	0.46***	0.51***	0.48***	0.58***	0.60***	1			
7. Command clarity	3.71	0.64	0.42***	0.45***	0.44***	0.51***	0.49***	0.53***	1		
8. Information flow efficiency	3.76	0.59	0.48***	0.50***	0.46***	0.54***	0.52***	0.56***	0.63***	1	
9. Interdepartmental trust	3.65	0.66	0.39***	0.42***	0.41***	0.47***	0.46***	0.49***	0.58***	0.61***	1

*** *p* < 0.001. Values below the diagonal represent Pearson correlation coefficients.

These findings indicate that the sample is representative of the hospital’s emergency response workforce and that the core constructs are moderately correlated yet empirically distinguishable, providing a suitable foundation for structural equation modeling.

### 3.2 Validation of the emergency risk governance capability measurement system

#### 3.2.1 Latent structure identification and measurement model fit

EFA results (Table 3): KMO = 0.924, Bartlett’s test *χ*^2^ = 5126.37 (df = 406, *p* < 0.001). Parallel analysis and scree plot supported a four-factor solution, cumulatively explaining 68.7% of variance (21.4, 18.3, 15.6, 13.4%), aligning with risk sensing, resource governance, collaborative governance, and dynamic resilience. Standardized loadings ranged 0.63–0.84 (*p* < 0.001). Primary-secondary loading differences >0.20, no notable cross-loadings.

**Table 3:** Exploratory factor analysis results.

Item	F1	F2	F3	F4	*h* ^2^	*u* ^2^
	Risk sensing	Resource governance	Collaborative network	Dynamic resilience		
RP1	0.78				0.64	0.36
RP2	0.81				0.68	0.32
RP3	0.74				0.61	0.39
RP4	0.69				0.56	0.44
RP5	0.76				0.63	0.37
RG1		0.75			0.59	0.41
RG2		0.82			0.7	0.3
RG3		0.78			0.65	0.35
RG4		0.73			0.57	0.43
RG5		0.8			0.68	0.32
RG6		0.71			0.54	0.46
CN1			0.77		0.6	0.4
CN2			0.83		0.72	0.28
CN3			0.79		0.66	0.34
CN4			0.74		0.58	0.42
CN5			0.81		0.69	0.31
DR1				0.76	0.58	0.42
DR2				0.82	0.71	0.29
DR3				0.78	0.64	0.36
DR4				0.74	0.55	0.45
DR5				0.8	0.68	0.32
DR6				0.73	0.57	0.43

Item codes: RP, risk sensing and interpretation; RG, resource governance capability; CN, collaborative governance network capability; DR, dynamic governance resilience. Empty cells indicate loadings <0.40.

Loadings <0.40 are suppressed. Communality (*h*^2^) and uniqueness (*u*^2^) are reported for each item.

**Figure 2:**
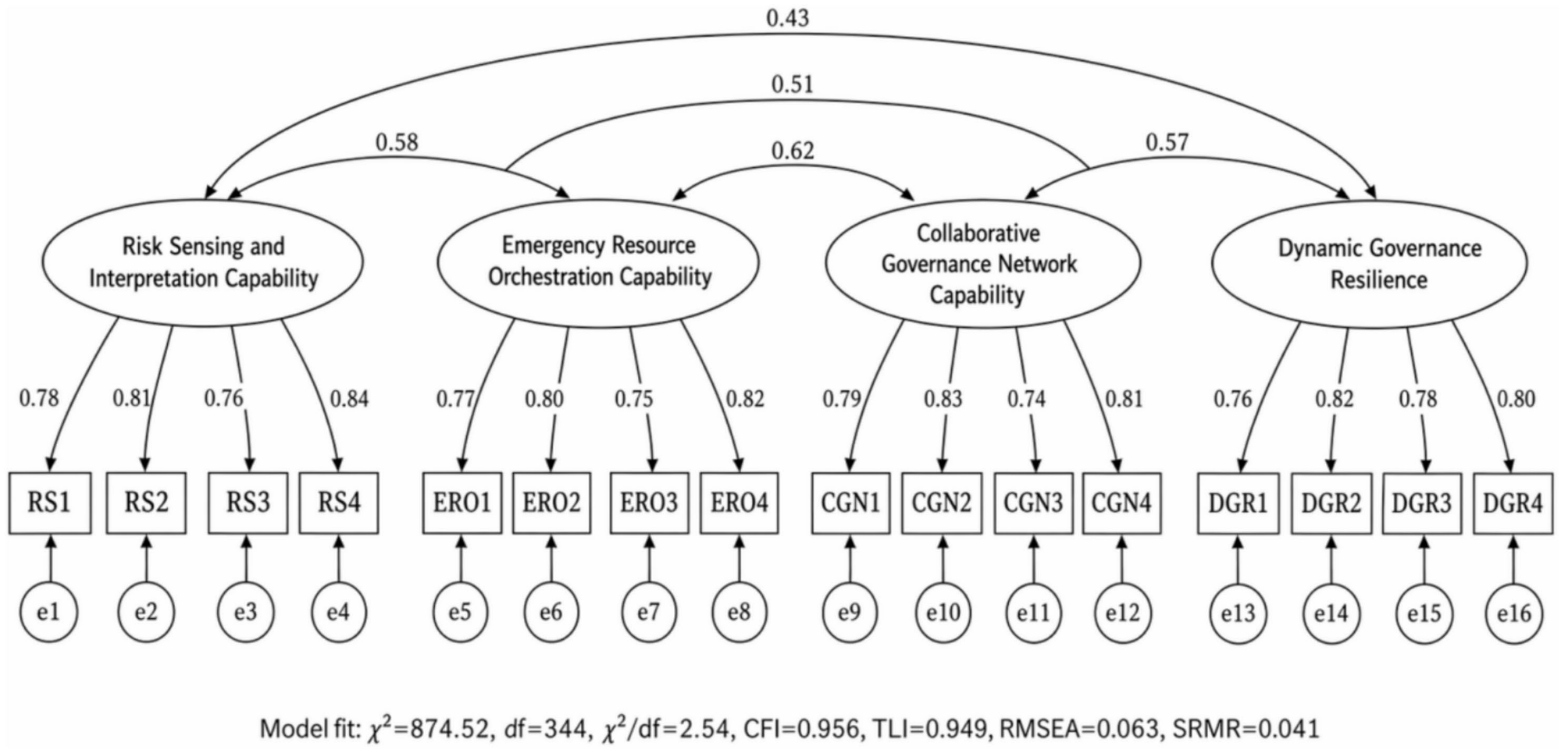
Four-factor confirmatory factor analysis model for the emergency risk governance capability measurement system. Ellipses represent latent constructs, rectangles represent observed indicators, and small circles represent residual terms. Values beside single-headed arrows are standardized factor loadings. Curved double-headed arrows represent latent-factor correlations. The model includes four distinct constructs: risk sensing and interpretation capability, emergency resource orchestration capability, collaborative governance network capability, and dynamic governance resilience. Model fit: *χ*^2^ = 874.52, df = 344, *χ*^2^/df = 2.54, CFI = 0.956, TLI = 0.949, RMSEA = 0.063, SRMR = 0.041.

CFA results (Figure 2) showed good fit for the four-factor measurement model (*χ*^2^ = 874.52, df = 344, *χ*^2^/df = 2.54, CFI = 0.956, TLI = 0.949, RMSEA = 0.063 [90% CI 0.058, 0.068], SRMR = 0.041). The figure presents four distinct latent constructs—risk sensing and interpretation capability, emergency resource orchestration capability, collaborative governance network capability, and dynamic governance resilience—consistent with the measurement design in Section 2.5.3. Standardized factor loadings were all >0.65 (*p* < 0.001), and inter-factor correlations were <0.80. A single-factor and two-factor model showed significantly worse fit (ΔCFI > 0.05, ΔRMSEA > 0.02), supporting the multidimensional structure.

#### 3.2.2 Reliability, convergent validity, and discriminant validity

Table 4 shows reliability and validity. Cronbach’s *α* ranged 0.87–0.90, CR 0.90–0.92, AVE 0.64–0.69. For discriminant validity: square root of each AVE (0.80–0.83) exceeded its highest inter-construct correlation (max *r* = 0.60–0.63) and exceeded MSV (0.38–0.45) and ASV (0.28–0.33). HTMT values <0.85. These findings support discriminant validity; the moderate correlation between resource orchestration and collaborative governance (*r* = 0.62) reflects conceptual adjacency.

**Table 4:** Reliability, convergent validity, and discriminant validity of constructs.

Construct	Items	Cronbach’s *α*	CR	AVE	√AVE	MSV	ASV	Max r
Risk sensing and interpretation capability	5	0.89	0.91	0.67	0.82	0.38	0.29	0.61
Resource governance capability	6	0.87	0.9	0.64	0.8	0.42	0.31	0.62
Collaborative governance network capability	5	0.88	0.91	0.66	0.81	0.4	0.28	0.63
Dynamic governance resilience	6	0.9	0.92	0.69	0.83	0.45	0.33	0.6

*α*, Cronbach’s alpha; CR, composite reliability; AVE, average variance extracted; √AVE, square root of AVE; MSV, maximum shared variance; ASV, average shared variance; Max *r*, maximum inter-construct correlation. Convergent validity is supported when CR > 0.70 and AVE > 0.50. Discriminant validity is supported when √AVE > MSV and √AVE > Max *r*.

These findings indicate that the measurement scales have high internal consistency, good convergent validity, and adequate discriminant validity, justifying their use in the structural model.

### 3.3 Structural evidence for hospital resilience formation

The structural model showed good fit (*χ*^2^ = 962.47, df = 382, *χ*^2^/df = 2.52, CFI = 0.953, TLI = 0.947, RMSEA = 0.062 [90% CI 0.057, 0.067], SRMR = 0.044; Table 5).

**Table 5:** Structural path estimates and control-variable effects (SEM).

Hypothesis	Path	B (unstd.)	SE	*β* (std.)	*z*/*t*	*p*	95% CI for *β*	*f* ^2^	Decision
Panel A. Structural path estimates									
H1	Risk sensing → resource governance	0.61	0.06	0.58	9.67	<0.001	[0.46, 0.69]	0.51	Supported
H2	Risk sensing → collaborative network	0.43	0.07	0.41	5.86	<0.001	[0.27, 0.54]	0.21	Supported
H3	Resource governance → collaborative network	0.55	0.05	0.52	10.4	<0.001	[0.42, 0.62]	0.38	Supported
H4	Resource governance → dynamic resilience	0.36	0.06	0.34	5.67	<0.001	[0.18, 0.48]	0.12	Supported
H5	Collaborative network → dynamic resilience	0.48	0.05	0.46	9.2	<0.001	[0.35, 0.56]	0.29	Supported
H6	Risk sensing → dynamic resilience	0.07	0.05	0.07	1.39	0.163	[−0.03, 0.17]	0.01	Not supported

Outcome variable	Control variable	Coding	B (unstd.)	SE	*β* (std.)	*z*/*t*	*p*	Interpretation
Panel B. Control-variable effects								
Risk sensing and interpretation capability	Gender	0 = male; 1 = female	0.04	0.04	0.05	0.98	0.327	Not significant
Risk sensing and interpretation capability	Age	1 = <25; 2 = 25–34; 3 = 35–44; 4 = ≥45	0.06	0.03	0.08	1.82	0.069	Positive, not significant
Risk sensing and interpretation capability	Education level	1 = junior college or below; 2 = bachelor; 3 = master or above	0.05	0.03	0.06	1.54	0.124	Not significant
Risk sensing and interpretation capability	Work tenure	1 = <5 years; 2 = 5–10; 3 = 11–20; 4 = > 20	0.03	0.03	0.04	1.11	0.267	Not significant
Risk sensing and interpretation capability	Administrative/support job type	0 = frontline clinical/nursing; 1 = administrative/support	−0.18	0.07	−0.15	−2.46	0.014	Administrative/support staff reported lower risk sensing than frontline staff
Emergency resource orchestration capability	Frontline care participation	0 = no; 1 = yes	0.16	0.06	0.17	2.66	0.008	Frontline staff reported stronger resource coordination capability
Emergency resource orchestration capability	Department risk level	0 = other units; 1 = high-risk units	0.12	0.05	0.11	2.21	0.028	High-risk units reported higher resource orchestration capability
Collaborative governance network capability	Frontline care participation	0 = no; 1 = yes	0.11	0.05	0.12	2.16	0.031	Frontline staff reported stronger collaborative network capability
Collaborative governance network capability	Management level	0 = general staff; 1 = middle/senior management	0.1	0.05	0.09	1.94	0.053	Marginal positive association
Dynamic governance resilience	Management level	0 = general staff; 1 = middle/senior management	0.14	0.06	0.14	2.35	0.019	Management staff reported higher resilience
Dynamic governance resilience	Age	1 = <25; 2 = 25–34; 3 = 35–44; 4 = ≥45	0.07	0.04	0.09	1.73	0.084	Positive, not significant
Dynamic governance resilience	Education level	1 = junior college or below; 2 = bachelor; 3 = master or above	0.06	0.04	0.07	1.59	0.112	Positive, not significant
Dynamic governance resilience	Work tenure	1 = <5 years; 2 = 5–10; 3 = 11–20; 4 = > 20	0.04	0.03	0.05	1.21	0.226	Not significant

B, unstandardized estimate; *β*, standardized estimate; CI, bias-corrected 95% confidence interval based on 5,000 bootstrap resamples; *f*^2^ indicates local effect size (0.02 = small, 0.15 = medium, 0.35 = large). All tests are two-tailed. Only control variables retained in the final parsimonious model are shown in Panel B; variables not reaching statistical significance across equations were omitted from the final displayed table for brevity, except for key demographic controls (gender, age, education, and work tenure), which are retained for interpretive completeness.

**Figure 3:**
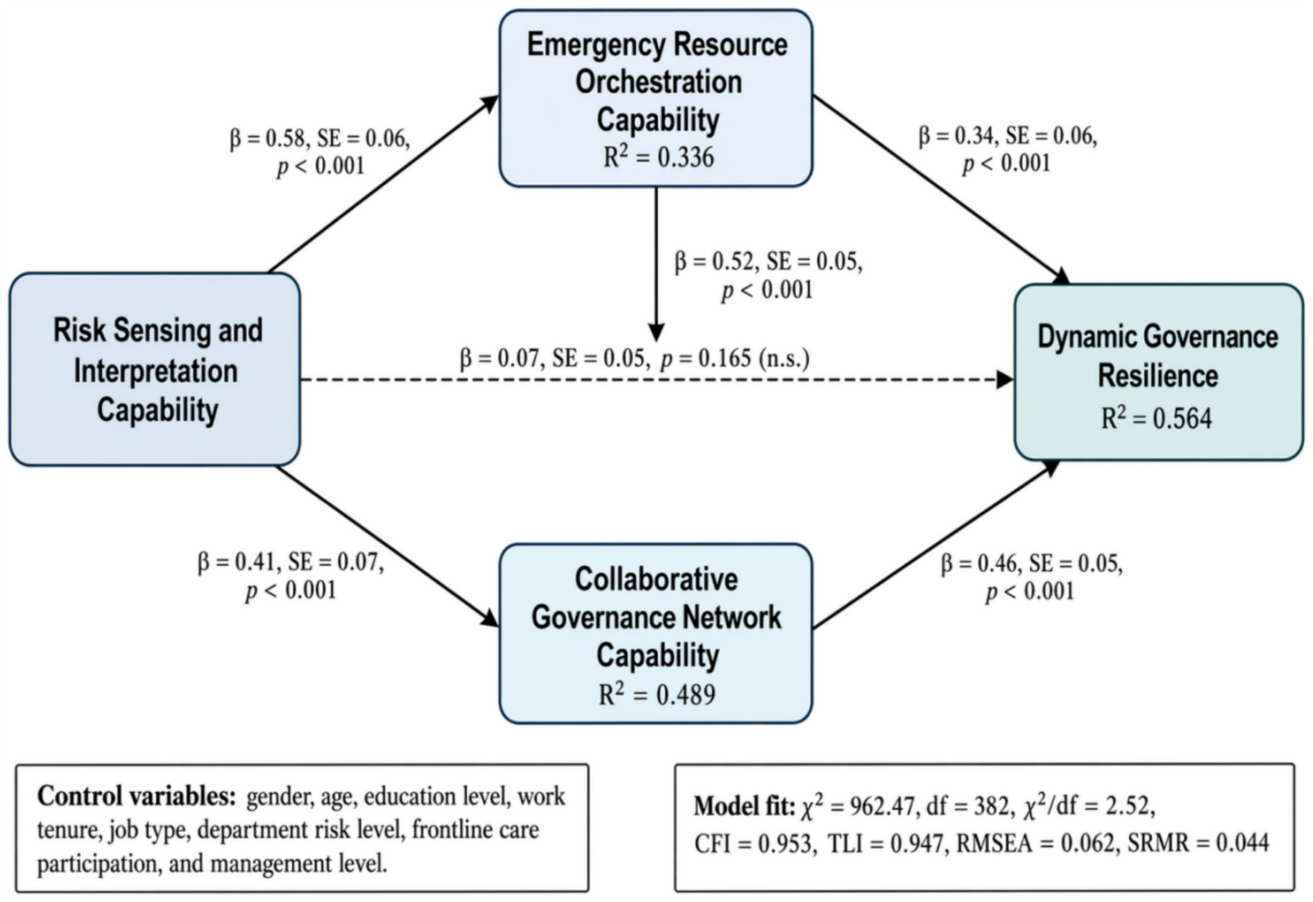
Structural equation model of hospital resilience formation. Values on solid arrows are standardized coefficients, with standard errors shown in parentheses. Dashed arrow indicates a non-significant direct path. *R*^2^ values are shown inside endogenous latent-variable nodes. Control variables were gender, age, education level, work tenure, job type, department risk level, frontline care participation, and management level. Panel B. Model fit: *χ*^2^ = 962.47, df = 382, *χ*^2^/df = 2.52, CFI = 0.953, TLI = 0.947, RMSEA = 0.062, SRMR = 0.044.

As shown in the Figure 3, risk sensing and interpretation capability was significantly associated with emergency resource orchestration capability (*β* = 0.58, SE = 0.06, *p* < 0.001) and collaborative governance network capability (*β* = 0.41, SE = 0.07, *p* < 0.001). Emergency resource orchestration capability was associated with Collaborative governance network capability (*β* = 0.52, SE = 0.05, *p* < 0.001). Emergency resource orchestration capability (*β* = 0.34, SE = 0.06, *p* < 0.001) and collaborative governance network capability (*β* = 0.46, SE = 0.05, *p* < 0.001) were both associated with dynamic governance resilience, with collaborative governance network capability showing a stronger association. The direct path from risk sensing and interpretation capability to dynamic governance resilience was non-significant after including mediators (*β* = 0.07, SE = 0.05, *p* = 0.163), indicating full mediation.

The model explained 33.6% of the variance in emergency resource orchestration capability (*R*^2^ = 0.336), 48.9% of the variance in collaborative governance network capability (*R*^2^ = 0.489), and 56.4% of the variance in dynamic governance resilience (*R*^2^ = 0.564).

Control variable effects (Table 5): Frontline care participation was positively associated with resource orchestration (*β* = 0.17, *p* = 0.008) and collaborative governance (*β* = 0.12, *p* = 0.031). Management level was positively associated with dynamic resilience (*β* = 0.14, *p* = 0.019). Administrative/support job type was negatively associated with risk sensing relative to frontline staff (*β* = −0.15, *p* = 0.014). Age (*β* = 0.09, *p* = 0.084) and education (*β* = 0.07, *p* = 0.112) were non-significant. Gender and work tenure showed no significant effects.

These findings indicate that hospital resilience is primarily driven by resource orchestration and collaborative governance, with risk sensing exerting its influence indirectly through these mediating mechanisms. Collaborative governance has the strongest direct association with resilience, highlighting the critical role of cross-departmental coordination.

### 3.4 Analysis of mediating transmission paths

**Figure 4:**
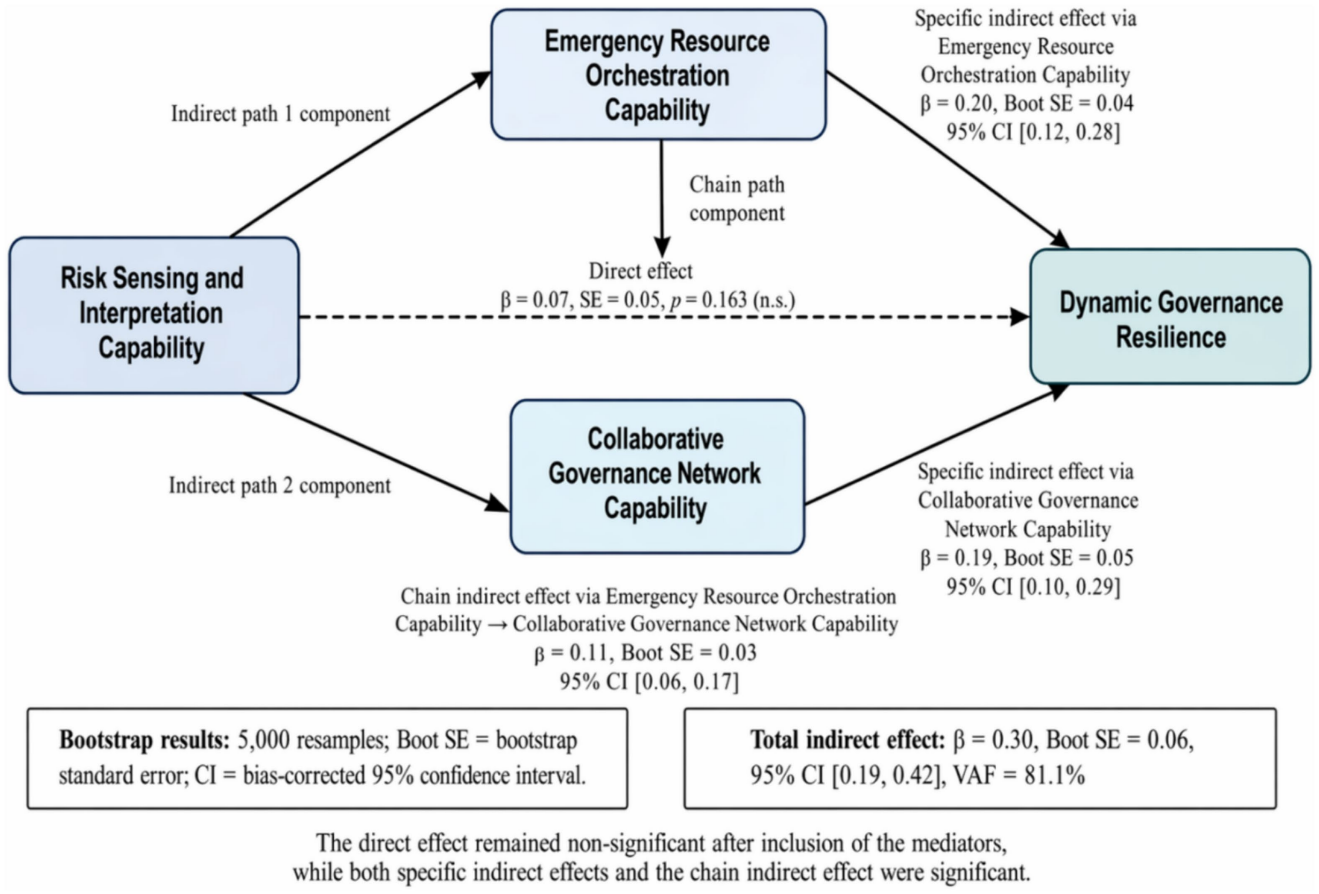
Bootstrap mediation decomposition of the effect of risk sensing and interpretation capability on dynamic governance resilience. Values are standardized effects estimated using 5,000 bootstrap resamples. Boot SE, bootstrap standard error; CI, bias-corrected 95% confidence interval. The direct effect remained non-significant after inclusion of emergency resource orchestration capability and collaborative governance network capability, whereas the two specific indirect effects and the chain indirect effect were significant.

Bootstrap results (5,000 resamples; Table 6) showed significant indirect effects. The specific indirect effect through emergency resource orchestration capability was significant (*β* = 0.20, Boot SE = 0.04, 95% CI [0.12, 0.28]). The specific indirect effect through collaborative governance network capability was also significant (*β* = 0.19, Boot SE = 0.05, 95% CI [0.10,0.29]). The chain-mediated pathway from risk sensing and interpretation capability to dynamic governance resilience through emergency resource orchestration capability and collaborative governance network capability was significant (*β* = 0.11, Boot SE = 0.03, 95% CI [0.06,0.17]). The total indirect effect was *β* = 0.30 (Boot SE = 0.06, 95% CI [0.19,0.42]), accounting for 81.1% of the total effect. The direct effect from risk sensing and interpretation capability to dynamic governance resilience remained non-significant after the mediators were included (*β* = 0.07, SE = 0.05, *p* = 0.163). These mediation pathways are illustrated in Figure 4.

**Table 6:** Bootstrap decomposition of direct, indirect, and total effects (5,000 resamples).

Effect type	From → To	Mediator pathway	B (unstd.)	*β* (std.)	Boot SE	*z*	*p*	BCa 95% CI (*β*)	Percentile 95% CI (*β*)	VAF (%)	Inference
Direct	RS → RG	—	0.61	0.58	0.06	9.67	<0.001	[0.46, 0.69]	[0.45, 0.68]	—	Significant
Direct	RS → CN	—	0.43	0.41	0.07	5.86	<0.001	[0.27, 0.54]	[0.26, 0.53]	—	Significant
Direct	RG → CN	—	0.55	0.52	0.05	10.4	<0.001	[0.42, 0.62]	[0.41, 0.61]	—	Significant
Direct	RG → DR	—	0.36	0.34	0.06	5.67	<0.001	[0.18, 0.48]	[0.17, 0.47]	—	Significant
Direct	CN → DR	—	0.48	0.46	0.05	9.2	<0.001	[0.35, 0.56]	[0.34, 0.55]	—	Significant
Direct	RS → DR	—	0.07	0.07	0.05	1.39	0.163	[−0.03, 0.17]	[−0.04, 0.16]	—	Not significant
Indirect (specific)	RS → DR	RS → RG → DR	0.22	0.2	0.04	5	<0.001	[0.12, 0.28]	[0.11, 0.27]	54.1	Significant
Indirect (specific)	RS → DR	RS → CN → DR	0.21	0.19	0.05	3.8	<0.001	[0.10, 0.29]	[0.09, 0.28]	51.4	Significant
Indirect (specific)	RS → DR	RS → RG → CN → DR	0.12	0.11	0.03	3.67	<0.001	[0.06, 0.17]	[0.05, 0.16]	29.7	Significant
Indirect (total)	RS → DR	RG, CN, RG → CN	0.33	0.3	0.06	5	<0.001	[0.19, 0.42]	[0.18, 0.41]	81.1	Significant
Total	RS → DR	Direct + indirect	0.4	0.37	0.07	5.29	<0.001	[0.23, 0.50]	[0.22, 0.49]	—	Significant
Indirect (specific)	RS → CN	RS → RG → CN	0.32	0.3	0.06	5	<0.001	[0.19, 0.42]	[0.18, 0.41]	42.3	Significant
Total	RS → CN	Direct + indirect	0.75	0.71	0.08	8.88	<0.001	[0.55, 0.82]	[0.54, 0.81]	—	Significant

RS, risk sensing; RG, resource governance; CN, collaborative network; DR, dynamic resilience. Bootstrapped SE, *z*, and *p* are based on 5,000 bias-corrected resamples. BCa CI, bias-corrected and accelerated confidence interval; percentile CI, percentile confidence interval. VAF (variance accounted for), total indirect effect/total effect × 100. Significance is inferred when the 95% CI does not include zero.

These findings indicate that risk sensing influences hospital resilience almost entirely through indirect pathways—first by enhancing resource orchestration, which in turn strengthens collaborative governance, and ultimately boosting resilience. The chain mediation model confirms the sequential governance logic proposed in the framework.

### 3.5 Heterogeneity across organizational subgroups

Measurement invariance was established across subgroups (Table 7). Configural invariance: CFI = 0.948–0.955, RMSEA = 0.060–0.065. Constraining factor loadings (ΔCFI = 0.004, ΔRMSEA = 0.002) and intercepts (ΔCFI = 0.006, ΔRMSEA = 0.003) yielded minimal fit deterioration.

**Table 7:** Multi-group structural path comparison across organizational subgroups.

Grouping variable	Path	Group 1 (*β*)	Group 2 (*β*)	Δ*β*	SE (Δ)	*z*-value	*p*-value	95% CI of Δ*β*	Conclusion
Frontline status	RS → RG	0.64***	0.49***	0.15	0.05	2.87	0.004	[0.05, 0.26]	Significant difference
Frontline status	RS → CN	0.43***	0.39***	0.04	0.04	1.02	0.308	[−0.04, 0.12]	No difference
Frontline status	RG → CN	0.53***	0.50***	0.03	0.04	0.75	0.454	[−0.05, 0.11]	No difference
Frontline status	CN → DR	0.47***	0.44***	0.03	0.04	0.81	0.418	[−0.05, 0.10]	No difference
Management level	RS → RG	0.59***	0.56***	0.03	0.05	0.61	0.542	[−0.07, 0.13]	No difference
Management level	RS → CN	0.42***	0.40***	0.02	0.04	0.5	0.615	[−0.06, 0.10]	No difference
Management level	CN → DR	0.53***	0.38***	0.15	0.06	2.54	0.011	[0.03, 0.27]	Significant difference
Management level	RG → DR	0.36***	0.33***	0.03	0.05	0.6	0.548	No difference	No difference
Department risk level	RG → CN	0.59***	0.44***	0.15	0.06	2.61	0.009	[0.04, 0.26]	Significant difference
Department risk level	RS → RG	0.61***	0.55***	0.06	0.05	1.2	0.23	[−0.04, 0.16]	No difference
Department risk level	RS → CN	0.41***	0.38***	0.03	0.04	0.75	0.453	[−0.05, 0.11]	No difference
Department risk level	CN → DR	0.48***	0.43***	0.05	0.05	1	0.317	[−0.05, 0.15]	No difference

RS, risk sensing; RG, resource governance; CN, collaborative network; DR, dynamic resilience. *β*, standardized path coefficient. Δβ represents cross-group differences. Cross-group differences in path coefficients were tested using *z*-tests. Significant differences are inferred when *p* < 0.05 and the 95% CI of Δ*β* does not include zero. *** *p* < 0.001.

By occupational type: Risk sensing → resource orchestration was stronger in frontline staff (*β* = 0.64) than non-frontline (*β* = 0.49, Δ*β* = 0.15, z = 2.87, *p* = 0.004).

By management level: Collaborative governance → dynamic resilience was stronger in middle/senior management (*β* = 0.53) than general staff (*β* = 0.38, Δ*β* = 0.15, z = 2.54, *p* = 0.011).

By department risk level: Resource orchestration → collaborative governance was stronger in high-risk departments (*β* = 0.59) than low-risk departments (*β* = 0.44, Δ*β* = 0.15, z = 2.61, *p* = 0.009). The direct path risk sensing → collaborative governance showed no significant subgroup differences.

These findings indicate that the governance pathways vary meaningfully across organizational roles and contexts. Frontline staff show a stronger link from risk sensing to resource mobilization, while managers rely more on collaborative governance to achieve resilience. High-risk departments benefit more from resource orchestration in fostering collaboration.

### 3.6 Robustness test results

Three competing models were tested (Table 8): Model A (parallel mediators), Model B (reversed mediation order), Model C (removed direct path). Main model fit (*χ*^2^/df = 2.52, CFI = 0.953, RMSEA = 0.062) was superior to Model A (*χ*^2^/df = 3.11, CFI = 0.921, RMSEA = 0.078), Model B (*χ*^2^/df = 3.26, CFI = 0.914, RMSEA = 0.081), and Model C (*χ*^2^/df = 2.95, CFI = 0.928, RMSEA = 0.074). Chi-square difference tests confirmed main model superiority (Δ*χ*^2^ = 86.74–142.31, Δdf = 3–5, *p* < 0.001).

**Table 8:** Comparison of competing structural models and common method bias models.

Model	Specification	*χ* ^2^	df	*χ*^2^/df	CFI	TLI	RMSEA	SRMR	AIC	Δ*χ*^2^	Δdf
M0	Baseline CFA (four-factor)	1214.36	410	2.96	0.931	0.924	0.071	0.058	54832.7	—	—
M1	Main serial mediation model	962.47	382	2.52	0.953	0.947	0.062	0.044	54211.4	—	—
M2	Parallel mediation model	1087.95	386	2.82	0.921	0.914	0.078	0.059	54586.9	125.48***	4
M3	Reversed mediation order	1134.78	387	2.93	0.914	0.906	0.081	0.062	54742.5	172.31***	5
M4	No direct path (RS → DR removed)	1020.7	385	2.65	0.928	0.921	0.074	0.051	54398.2	58.23***	3
M5	Single-factor model (common method bias check)	2148.63	405	5.31	0.741	0.719	0.128	0.109	55896.4	1186.16***	23
M6	Four-factor model + latent method factor	901.84	358	2.52	0.958	0.951	0.057	0.041	54186.3	60.63***	24

M1 represents the hypothesized serial mediation model. M2 specifies resource governance and collaborative network as parallel mediators. M3 reverses the mediation order. M4 removes the direct path from risk sensing to dynamic resilience. M5 is Harman-style single-factor/common-method comparison model. M6 adds an unmeasured latent method factor loading on all indicators to evaluate potential common method bias. Δ*χ*^2^ values are calculated relative to the main structural model (M1), except where model structure indicates a specific CMB comparison. *** *p* < 0.001.

Common method bias diagnostics (Tables 8, 9, Panel A): Harman’s single-factor test: first unrotated factor accounted for 31.8% of variance (below 40%). Full-collinearity VIF values ranged 1.82–2.64 (below 3.3). Adding a latent method factor yielded minimal fit improvement (ΔCFI = 0.005, ΔRMSEA = −0.005) and path coefficient changes of 0.02–0.05.

**Table 9:** Common method bias diagnostics and robustness tests based on multi-source data and alternative estimation strategies.

Panel A. Common method bias diagnostics				
Dererriagnostic	Indicator/Comparison	Result	Criterion/Reference	Interpretation
Harman’s single-factor test	Variance explained by first unrotated factor	31.80%	<40%	No evidence of severe common method bias
Full-collinearity assessment	Minimum VIF across core latent constructs	1.82	<3.3	Acceptable
Full-collinearity assessment	Maximum VIF across core latent constructs	2.64	<3.3	Acceptable
Latent method factor comparison	ΔCFI (M6 vs. M1)	0.005	Small change	Limited incremental improvement
Latent method factor comparison	ΔRMSEA (M6 vs. M1)	−0.005	Small change	Limited incremental improvement
Latent method factor comparison	Range of Δ*β* for core paths	0.02–0.05	Minimal change	Main conclusions unchanged
Overall judgment	Combined evidence	—	—	CMB cannot be fully excluded, but is unlikely to materially distort substantive conclusions

Panel B. Robustness tests based on multi-source data and alternative estimation strategies										
Path	Main model	Archival Proxy 1 (material turnover)	Archival Proxy 2 (training coverage)	Archival Proxy 3 (meeting frequency)	MLR estimator	WLSMV estimator	Frontline subsample	Management subsample	Trimmed sample (5%)	Stability assessment
RS → RG	0.58***	0.55***	0.57***	0.54***	0.57***	0.59***	0.55***	0.61***	0.56***	Stable
RS → CN	0.41***	0.39***	0.42***	0.40***	0.39***	0.43***	0.38***	0.44***	0.40***	Stable
RG → CN	0.52***	0.50***	0.53***	0.49***	0.51***	0.54***	0.48***	0.55***	0.51***	Stable
RG → DR	0.34***	0.32***	0.36***	0.33***	0.32***	0.35***	0.30***	0.37***	0.33***	Stable
CN → DR	0.46***	0.44***	0.47***	0.45***	0.44***	0.48***	0.42***	0.49***	0.45***	Stable
RS → DR	0.07 (ns)	0.06 (ns)	0.08 (ns)	0.05 (ns)	0.05 (ns)	0.09 (ns)	0.04 (ns)	0.10 (ns)	0.06 (ns)	Not significant

Panel A summarizes common method bias diagnostics. Harman’s single-factor test reports the variance explained by the first unrotated factor. VIF, full-collinearity variance inflation factor calculated for the core latent constructs. Latent method factor comparison refers to the model that adds an unmeasured common latent factor to all observed indicators. Panel B reports standardized path coefficients (*β*). Archival proxy models replace subjective measures with objective management indicators. MLR, robust maximum likelihood; WLSMV, weighted least squares mean- and variance-adjusted. Trimmed sample excludes the upper and lower 5% of observations. *** *p* < 0.001; ns, not significant.

**Figure 5:**
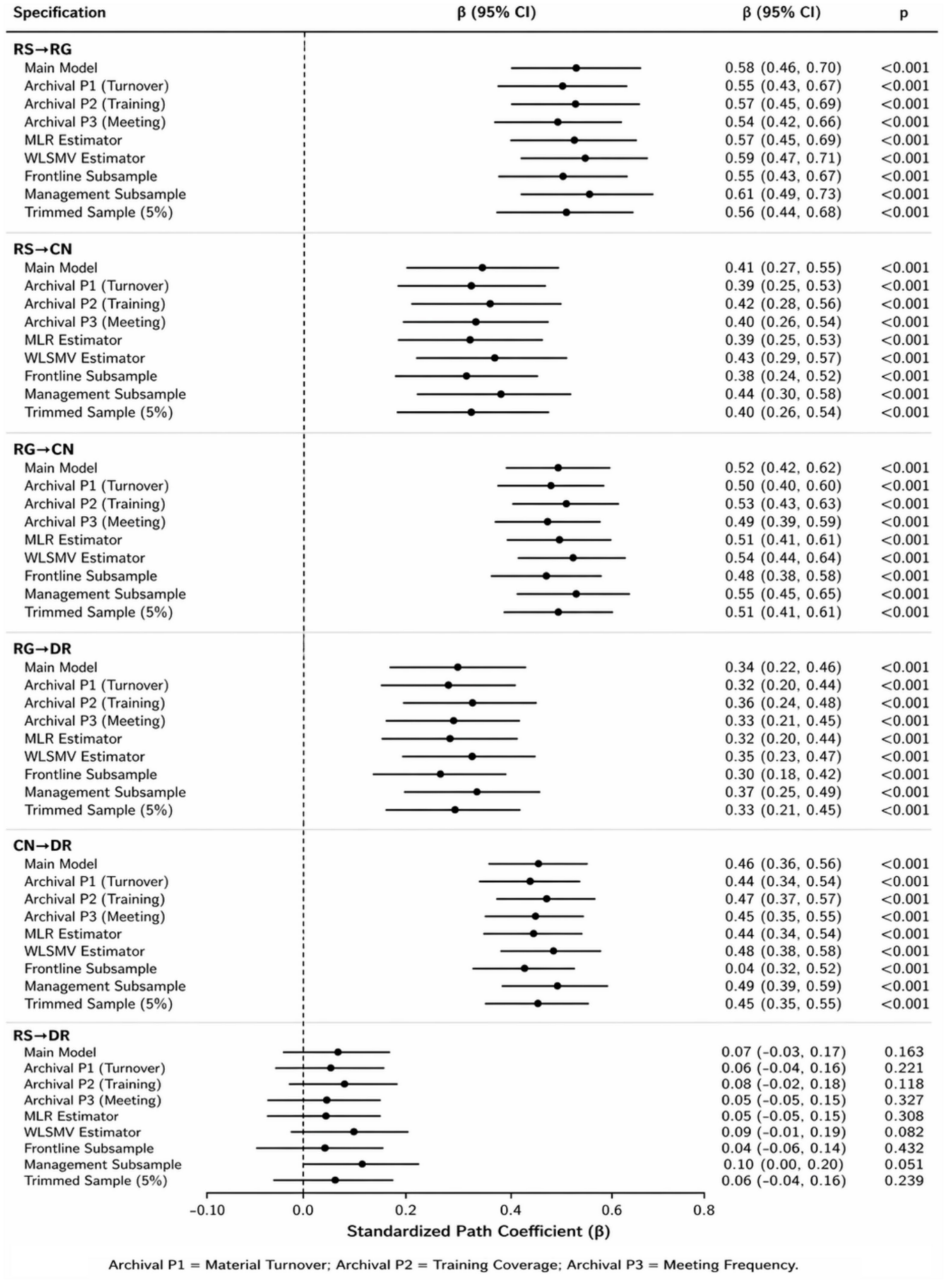
Robustness of key structural paths across multi-source and alternative estimation specifications. Points represent standardized path coefficients (*β*). Horizontal bars represent 95% confidence intervals. The vertical dashed line marks *β* = 0. Specifications correspond to Table 9. Panel B: Main model; archival proxy 1 (material turnover); archival proxy 2 (training coverage); archival proxy 3 (meeting frequency); MLR estimator; WLSMV estimator; frontline subsample; management subsample; and trimmed sample (5%). RS, risk sensing and interpretation capability; RG, emergency resource orchestration capability; CN, collaborative governance network capability; DR, dynamic governance resilience.

Multi-source robustness (Table 9, Panel B; Figure 5): Replacing subjective indicators with objective archival proxies (material turnover efficiency, training coverage, meeting frequency) yielded consistent path estimates. Core path standardized coefficients remained stable across the main model, archival proxy models, alternative estimators (MLR and WLSMV), and sample specifications (frontline subsample, management subsample, and trimmed sample). The direct path from Risk sensing and interpretation capability to dynamic governance resilience remained non-significant across all specifications. Additional robustness details are in Supplementary Appendix Section S8.

These findings indicate that the hypothesized serial mediation model is the most appropriate specification among the alternatives, and that the results are robust against common method bias, alternative estimation techniques, and different sample compositions. The core structural relationships are not artifacts of measurement or model choice.

## 4 Discussion

Public health emergencies are becoming more frequent and often occur together. This trend amplifies the uncertainty and vulnerability of hospital systems. In response, this study examined how hospital resilience is generated. We established a structural chain of influence among three governance capabilities: risk perception, resource preparedness and governance, and team collaboration. We then used structural equation modeling to test direct, mediating, and chain mediation effects at the same time. This allowed us to explain the organizational reasons why hospitals perform differently under similar shocks. The main model fit the data well, and key pathways remained consistent across robustness checks. These results support the view that hospital resilience in this setting is closely linked to governance processes, not to singular resource stockpiles. However, because the data came from a single highly resourced tertiary hospital, the findings should be interpreted primarily as evidence from a large, institutionally mature hospital context.

From a theoretical perspective, the present study contributes in several ways. First, it connects hospital resilience research with the foundational dynamic capability tradition. Teece et al. (9) define dynamic capabilities as the capacity to integrate, build, and reconfigure internal and external competences in changing environments. Teece (10) later specifies this logic through the microfoundations of sensing, seizing, and reconfiguring. Eisenhardt and Martin (11) complement this view by treating dynamic capabilities as identifiable organizational processes that enable resource recombination under uncertainty. Second, this study tests a serial mediation mechanism rather than only direct paths, thereby explaining how risk sensing may be translated into resilience through resource orchestration and collaborative governance. Third, it frames hospital resilience as a governance-capability construct rather than merely an outcome. This framing is consistent with post-2010 health-system resilience scholarship: Kruk et al. (4) emphasize the capacity of health systems to absorb disturbance while maintaining core functions, and Blanchet et al. (5) further articulate resilience in terms of absorptive, adaptive, and transformative capacities. Barasa et al. (6) also support treating resilience as an emergent property of complex health systems rather than a single static outcome. Fourth, the study integrates risk sensing, resource orchestration, and collaborative governance within a single structural framework. In doing so, it responds to empirical and European policy calls for more context-sensitive and multi-level resilience analysis, including broader empirical reviews of health-system resilience and European Observatory work on resilience across preparedness, shock management, recovery, and learning phases (7, 18).

We found that risk perception was positively linked to resource preparedness and governance capability, and also to stronger collaborative networks. However, when we added these mediators, the direct link between risk perception and resilience became non-significant. This suggests that risk perception acts mainly as an early trigger in the emergency governance chain, rather than being sufficient to create resilience by itself. This finding adds to evidence about healthcare workers’ workplace preparedness during pandemics. Earlier research in the US Veterans Health Administration system showed that workers felt more prepared when they had organizational support, proper training, and a clear role (21). Thus, individual preparedness only helps when it is embedded in institutional systems and response processes. Another SEM study found that risk perception can directly affect a worker’s personal resilience (22). Why did we not see a direct effect at the organizational level? The reason may be that individual and organizational resilience mean different things. Individual resilience is about coping with stress and managing emotions, whereas hospital resilience is about keeping critical functions running and recovering from shocks. To bridge this gap, hospitals need an institutional process that turns awareness into action, a process that requires resource orchestration and cross-unit teamwork.

Within the pathway linking risk perception to resource governance, our study highlights the value of governance-oriented resources. Resource governance not only directly improves resilience but also strengthens collaborative networks, thus playing a key role in generating resilience. This finding matches multi-center hospital disaster assessments using the WHO Hospital Safety Index. Those assessments show that preparedness varies across structural, non-structural, and functional domains, and that functional elements like emergency planning and coordination remain important for response capability (23). Similarly, action research using the FOCUS-PDCA model shows that hospitals can improve their non-structural preparedness through structured quality improvement tools and inter-departmental collaboration (24). This implies that changes in governance mechanisms can raise a system’s load-bearing capacity without adding many new supplies. From a supply perspective, system dynamics research has modeled purchasing and supply strategies for PPE and ventilators during pandemics. The findings indicate that direct tenders, stockpiling, domestic production, support for innovative supply arrangements, and ventilator sharing all help health systems prepare and respond (25). This fits with our approach of treating resource preparedness as an orchestration and governance capability, rather than just a stockpile.

Compared with resource governance, team collaboration showed a stronger positive association with hospital resilience and had a stable direct effect in our structural model. This suggests that collaborative networks may act as a critical amplifier, turning resources into sustained service delivery capacity. This result aligns closely with team training intervention research. For instance, TeamSTEPPS training significantly improved teamwork perceptions and patient safety culture among newly graduated hospital nurses (17). This shows that collaborative capability can be shaped and internalized as organizational routines through standardized training. Furthermore, a prospective randomized controlled trial found that contextualized in-situ simulation training during the pandemic improved interprofessional communication, skills, and teamwork when transferring critically ill COVID-19 patients (26). This provides stronger evidence that collaborative capability can be improved. Meanwhile, qualitative research on healthcare team resilience during COVID-19 viewed the team as a collective cognitive entity, highlighting anticipation, adaptation, cohesion, shared responsibility, communication, and transferable skills as mechanisms that shape resilient performance (27). This matches our finding that collaborative networks contribute significantly to resilience.

Our mediation and chain mediation results further clarify the step-by-step logic of the governance process. Specifically, risk perception is fully mediated by resource governance and collaborative networks in its effect on resilience. There is a clear chain: resource governance fosters collaboration, and collaboration then elevates resilience. This indicates that resilience is not determined by any single capability but emerges from the coupling and sequential interplay of multiple capabilities. Qualitative research on hospital functional preparedness similarly identifies leadership, planning, training and exercises, and communication or coordination as important categories of disaster preparedness (28). These categories are best interpreted as interrelated components rather than isolated inputs. In more structured hospital-level assessments, a cross-sectional survey of all hospitals in one region used factor analysis to validate a multi-dimensional disaster preparedness framework, identifying management, communication, and training-related factors as important contributors (29). This resonates with our integration of resource governance and collaborative networks into a unified structural framework and our identification of chain effects.

These comparisons also reveal that much existing research has remained at the stage of identifying preparedness dimensions or performing correlational analysis. By estimating direct and indirect effects simultaneously, our study provides structural evidence for how risk perception is institutionally translated into resilience performance, thereby improving the replicability of mechanistic explanations. At the same time, broader empirical reviews show that health-system resilience research remains sensitive to conceptual framing, measurement choices, and system context (18). European policy work further situates resilience across preparedness, shock management, recovery, and learning phases, underscoring the importance of governance and policy coordination across the shock cycle (7). Comparative European evidence from the first wave of COVID-19 shows that hospital resilience depended not only on pre-pandemic care structures and bed capacity but also on how hospitals and health systems managed capacity expansion and service reorganization during the crisis (17). Therefore, our single-hospital model should be understood as a mechanism-oriented contribution that requires further testing in European, multi-hospital, and cross-institutional contexts.

Regarding heterogeneous findings, we found that the driving effect of risk perception on resource governance was stronger among frontline positions. In contrast, the contribution of collaborative networks to resilience was higher among management positions. This suggests that a person’s location within the organization may alter key nodes in the governance chain, due to differences in information exposure and decision rights. Cross-regional surveys of healthcare workers show that perceptions of hospital preparedness during COVID-19 varied across economically diverse regions and were related to factors such as training exposure and institutional context (30). This helps explain why subgroup differences in information and role responsibilities may shape the governance pathways we observed. Frontline personnel typically encounter unusual signals earlier and experience resource shortages more directly, so their risk perception is more likely to trigger resource mobilization imperatives. In contrast, management positions bear responsibility for cross-unit coordination and command, making their performance more dependent on whether collaborative governance structures operate effectively. This interpretation is consistent with our control variable results, which showed that frontline participation was positively associated with resource orchestration and collaborative governance, whereas management level was positively associated with dynamic governance resilience. Research on rural hospital incident command leaders similarly indicates that geographic isolation, resource constraints, and limited disaster-response experience are seen as barriers to command coordination and response confidence (31). This further suggests that differences in resilience may stem less from resource volume alone than from whether collaborative structures can sustain effective operation under adverse conditions.

The robustness of our study derives from multiple factors, including good overall model fit, weaker explanatory power of alternative models, and consistent direction of key pathways across different specifications. This matches recent efforts to construct and validate organizational resilience models for hospitals in emergencies and disasters, which have also reported satisfactory model fit (32). Nonetheless, potential biases warrant careful attention. First, most of our variables come from surveys and retrospective integration. Although we conducted multiple statistical checks—including Harman’s single-factor test, full-collinearity diagnostics, and latent method factor comparison—we cannot fully exclude the possibility of common method bias and social desirability bias. This is especially relevant because several core constructs, such as risk perception, collaboration, and resilience, were assessed using self-reported Likert-scale measures. Additionally, our study was anchored in the hospital’s response to the initial COVID-19 outbreak and relied in part on retrospective assessments of that period. Accordingly, the findings should be interpreted with possible recall bias and the exceptional institutional conditions of that specific emergency in mind. Research on nurses’ household emergency preparedness found that perceived preparedness and actual preparedness were related but not identical, and that their associated factors differed (33). Although this evidence concerns household rather than organizational preparedness, it cautions against treating self-reported preparedness measures as fully equivalent to objective preparedness or performance. Second, the path strengths we identified may be influenced by inter-institutional variations in culture and governance structure. Future research should replicate our findings across regions and multi-center settings, incorporating objective operational data to strengthen causal inference and external validity. Thus, our reported associations are robust but may still be affected by shared measurement context. Future studies should further strengthen design-based controls by adding more objective operational indicators, multi-informant assessments, and longitudinal data.

From a practical standpoint, our study supports building resilience through actionable governance levers. Risk perception should be treated as an organizational capability rather than just an individual attitude. Standardized risk communication, situational assessment meetings, and early warning mechanisms should be used to enhance interpretive consistency, and these should then be embedded into resource mobilization protocols and authorization systems. Resource preparedness needs to shift from inventory management to orchestration and governance, using scenario-based demand forecasting and dynamic dispatch mechanisms to improve resource availability under peak loads. Research on machine learning-based emergency department crowding prediction shows that integrating operational and contextual data can yield predictive tools to support resource planning and earlier interventions (34), offering a technological pathway from risk identification to resource governance. Collaborative network development should focus on role clarity, information flow, and interprofessional training. It should institutionalize collaborative routines through frequent, low-cost drills and debriefings, and should also establish a command structure within management that can coordinate across units. This would help reduce the risk of ad-hoc coordination failures. When facing multi-source, composite shocks, digital infrastructure and business continuity must be included in the resilience governance remit. Research on US hospitals, clinics, and other healthcare delivery organizations indicates that ransomware attacks increased between 2016 and 2021, often accompanied by electronic system downtime and appointment cancellations (35). Such disruptions can also affect emergency department visits and inpatient admissions at targeted and nearby hospitals (36). This signals that the hospital resilience agenda must expand to include alternative processes and inter-institutional collaboration arrangements for cyber incidents and information system shocks.

This study has several limitations. First, because the analysis relied on retrospective, cross-sectional data from a single institution, the observed associations cannot definitively rule out endogeneity, reverse causality, or omitted variable bias, despite multiple robustness checks. It is possible, for example, that hospitals with stronger resilience are also better able to organize resources and sustain collaborative governance. In addition, resilience was measured mainly through perceptual and process-oriented indicators and was not yet systematically linked to objective outcomes such as service continuity performance, patient outcomes, or information system availability. Second, the study was conducted in a single large tertiary Grade A hospital in Beijing. Although this setting is informative for understanding governance mechanisms in a highly resourced hospital with relatively mature organizational systems, the findings may not transfer directly to primary hospitals, rural institutions, or resource-constrained settings. Broader empirical reviews indicate that health-system resilience findings are sensitive to system context, study design, and measurement choices (18). European policy work further emphasizes that resilience depends on governance across preparedness, shock management, recovery, and learning phases (7). In addition, comparative evidence from five countries during the first wave of COVID-19 shows that hospital care structures and capacity-expansion strategies shaped how hospitals coped with crisis conditions (17). Future research should therefore compare the proposed mechanism across hospitals embedded in different institutional systems, including European settings where resilience has been analyzed through care structures, surge capacity, policy coordination, and health-system recovery strategies. This boundary condition is particularly important because our study took place during the initial COVID-19 outbreak, an extraordinary institutional environment. In this context, the observed resilience may have been amplified by strong government coordination, emergency policy support, and broader system-level resource concentration. Third, although our discriminant validity tests met conventional thresholds, the correlation between Emergency Resource Orchestration Capability and Collaborative Governance Network Capability remained moderately high. This suggests that these two constructs are empirically distinguishable but conceptually adjacent. Future research should therefore use longitudinal, multi-wave, and multi-hospital comparative designs. It should also combine objective operational indicators with perceptual measures. Finally, researchers should test how governance mechanisms can be improved through intervention-oriented studies in a wider range of crisis contexts. To further support transparency and reproducibility, the revised manuscript includes a Supplementary Appendix. This appendix reports the bilingual disclosure version of the questionnaire items, construct definitions, variable coding, sample screening flow, ethical information, and additional robustness and bias diagnostics.

## 5 Conclusion

Based on a systematic retrospective analysis and structural modeling of hospital emergency operations during public health emergencies, this study examined how risk perception, resource preparedness, and team collaboration affect hospital resilience. We revealed the operational structure and transmission logic of emergency risk governance capabilities at the organizational level. The findings show that hospital resilience does not come from single resource inputs or temporary mobilization. Instead, it is built through the coordinated integration of risk identification, resource orchestration, and collaborative execution.

Risk sensing establishes the cognitive and institutional foundation for emergency response by improving resource governance and collaborative networks. Resource orchestration directly raises resilience and also strengthens cross-departmental collaboration, playing a key mediating role. Collaborative governance has the strongest effect on operational stability and functional reconfiguration. Mediation analysis further shows that risk sensing affects resilience mainly through the sequential pathways of resource governance and collaborative networks, displaying full mediation. These results provide empirical evidence for understanding how hospital resilience is generated.

Several limitations remain. First, our sample came from a single large public hospital, so external validity requires testing across multiple regions and institution types. Second, the retrospective design and subjective measures may introduce recall bias and context reconstruction errors. Third, we focused mainly on intra-organizational governance, with insufficient analysis of cross-institutional collaboration and regional governance structures. Future research should use multi-center comparative and longitudinal designs to test the model’s dynamic stability, situate hospitals within regional health governance systems, and integrate policy pilots to evaluate real-world interventions. This will enhance the value of our findings for public health emergency governance decision-making.

## Data Availability

The raw data supporting the conclusions of this article will be made available by the authors, without undue reservation.
