# Supplementary material for: Analysis of hospital resilience in public health emergencies using structural equation modeling: based on risk perception, resource preparedness, and team collaboration
Source: Front Public Health. 2026 May 15;14:1819684. doi: 10.3389/fpubh.2026.1819684 (PMC13219275; doi:10.3389/fpubh.2026.1819684)
Supplement: Supplementary file 1 [file Supplementary_file_1.docx]

Supplementary Appendix

**Analysis of Hospital Resilience in Public Health Emergencies Using Structural Equation Modeling**

*Prepared for manuscript revision; illustrative supplementary material for reviewer response*

# S1. Purpose and scope

This appendix consolidates the reproducibility information requested by reviewers, including the bilingual questionnaire items, construct definitions, item sources, response anchors, variable coding, sample screening flow, ethical information, and additional robustness / bias diagnostics.

# S2. Questionnaire administration and scoring

All survey items were administered as retrospective evaluations anchored to the hospital’s COVID-19 response from January to June 2020. Unless otherwise noted, perceptual items were scored on a 5-point Likert scale: 1 = Strongly disagree, 2 = Disagree, 3 = Neutral, 4 = Agree, and 5 = Strongly agree. Higher scores indicate stronger capability on the corresponding latent construct.

The final illustrative questionnaire contained 16 focal items across four latent constructs (4 items per construct), plus control-variable questions on demographic and work-related characteristics.

# S3. Bilingual questionnaire items and item sources

Table S1 presents the core bilingual items used in the revised manuscript. Item wording is shown as a contextually adapted and reviewer-oriented disclosure version. Sources indicate the theoretical origin of the item pool before translation, back-translation, expert revision, and contextual adaptation.

## Source legend:

- A = adapted from disaster risk perception / preparedness literature and emergency management capability scales.
- B = adapted from dynamic capability / resource orchestration literature and hospital emergency resource management practice.
- C = adapted from collaborative governance / interprofessional teamwork literature.
- D = adapted from organizational resilience / hospital resilience literature.

| **Construct** | **Code** | **English item** | **Chinese item** | **Source** | **Scale** |
| --- | --- | --- | --- | --- | --- |
| Risk Sensing and Interpretation Capability | RS1 | Our hospital was able to detect abnormal risk signals early during the COVID-19 response period. | 在新冠疫情应对期间，我院能够较早识别异常风险信号。 | A | 1–5 |
| Risk Sensing and Interpretation Capability | RS2 | Different departments had a largely consistent understanding of the urgency and severity of the emerging risk. | 不同部门对新发风险的紧迫性和严重程度基本形成了一致判断。 | A | 1–5 |
| Risk Sensing and Interpretation Capability | RS3 | The hospital translated emerging risk information into management action in a timely manner. | 医院能够将新出现的风险信息及时转化为管理行动。 | A | 1–5 |
| Risk Sensing and Interpretation Capability | RS4 | Even when information was incomplete, the hospital could make workable risk judgments and adjustments. | 即使在信息不完整的情况下，医院也能作出可操作的风险判断并及时调整。 | A | 1–5 |
| Emergency Resource Orchestration Capability | RG1 | Critical staff, supplies, and space could be redeployed across departments when needed. | 在需要时，关键人员、物资和场地能够在部门之间被快速调配。 | B | 1–5 |
| Emergency Resource Orchestration Capability | RG2 | The hospital had backup arrangements for essential supplies, processes, or functions. | 医院对关键物资、流程或功能具有备用安排。 | B | 1–5 |
| Emergency Resource Orchestration Capability | RG3 | Resources could be reconfigured quickly when operational demands changed. | 当运行需求变化时，医院能够较快重组相关资源。 | B | 1–5 |
| Emergency Resource Orchestration Capability | RG4 | Resource-allocation decisions were well integrated with the emergency command system. | 资源配置决策与应急指挥体系之间具有较强的嵌入性。 | B | 1–5 |
| Collaborative Governance Network Capability | CN1 | Authority and responsibilities across departments were clear during the emergency response. | 在应急响应过程中，跨部门的职责和权限界面比较清晰。 | C | 1–5 |
| Collaborative Governance Network Capability | CN2 | Information flowed quickly and accurately across the units involved in the response. | 参与应急响应的各单元之间信息传递及时且准确。 | C | 1–5 |
| Collaborative Governance Network Capability | CN3 | Departments maintained a workable level of mutual trust under high pressure. | 在高压情境下，各部门之间能够保持基本有效的相互信任。 | C | 1–5 |
| Collaborative Governance Network Capability | CN4 | The hospital was able to adjust collaboration arrangements as the situation evolved. | 随着情势变化，医院能够及时调整协作方式。 | C | 1–5 |
| Dynamic Governance Resilience | DR1 | The hospital was able to maintain critical services under peak pressure. | 在高峰压力下，医院能够维持关键服务的连续性。 | D | 1–5 |
| Dynamic Governance Resilience | DR2 | The hospital could reorganize functions and workflows when normal operations were disrupted. | 当常规运行受扰时，医院能够重组功能与工作流程。 | D | 1–5 |
| Dynamic Governance Resilience | DR3 | After the emergency phase, the hospital translated lessons learned into process or policy improvement. | 在应急阶段后，医院能够将经验教训转化为流程或制度改进。 | D | 1–5 |
| Dynamic Governance Resilience | DR4 | The hospital retained organizational memory that could support future emergency response. | 医院能够保留支持未来应急响应的组织记忆。 | D | 1–5 |

*Table S1. Bilingual disclosure version of the focal questionnaire items used in the revised manuscript.*

# S4. Construct definitions and measurement intent

| **Construct** | **Definition used in the manuscript** |
| --- | --- |
| Risk Sensing and Interpretation Capability | The organizational capability to identify, interpret, and act upon emerging risk information in a timely and internally consistent manner. |
| Emergency Resource Orchestration Capability | The organizational capability to allocate, redeploy, and reconfigure staff, materials, space, and procedures under emergency conditions. |
| Collaborative Governance Network Capability | The organizational capability to sustain clear command interfaces, information flow, trust, and adaptive cross-departmental coordination. |
| Dynamic Governance Resilience | The organizational capability to absorb shocks, maintain critical functions, reorganize operations, and institutionalize learning after disruption. |

*Table S2. Construct definitions used to support item disclosure and conceptual reproducibility.*

# S5. Control variables and coding scheme

| **Variable** | **Coding** | **Role in model** |
| --- | --- | --- |
| Gender | 0 = male; 1 = female | Covariate |
| Age group | 1 = <25 years; 2 = 25–34 years; 3 = 35–44 years; 4 = ≥45 years | Ordered categorical |
| Education level | 1 = junior college or below; 2 = bachelor; 3 = master or above | Ordered categorical |
| Work tenure | 1 = <5 years; 2 = 5–10 years; 3 = 11–20 years; 4 = >20 years | Ordered categorical |
| Administrative/support job type | 0 = frontline clinical/nursing; 1 = administrative/support | Dummy-coded comparison |
| Department risk level | 0 = other units; 1 = high-risk units | Dummy-coded comparison |
| Frontline care participation | 0 = no; 1 = yes | Dummy-coded comparison |
| Management level | 0 = general staff; 1 = middle/senior management | Dummy-coded comparison |

*Table S3. Control-variable coding scheme reported in the revised Methods and Results sections.*

# S6. Sample screening flow

The formal questionnaire returned 428 complete submissions. Cases were screened sequentially for severe missingness, response-pattern anomalies, logical inconsistency, and insufficient evidence of independent completion. A total of 42 cases were excluded, yielding 386 valid responses (effective response rate: 90.2%).

1. Returned questionnaires: 428
2. Removed for abnormal response duration / low-quality completion signals: 15
3. Removed for repetitive or homogeneous response patterns: 11
4. Removed for logical inconsistency in key items: 9
5. Removed for excessive missingness or unusable linkage information: 7
6. Final analytic sample: 386

# S7. Ethical approval and data governance

The study was reviewed and approved by the Ethics Committee of Beijing Jishuitan Hospital (Approval No. 2020-K-042-01) prior to the commencement of data collection. In accordance with the approved protocol, informed consent was obtained from all individual participants included in the study. For the questionnaire component, a detailed study information statement was presented on the survey homepage, and participants provided explicit consent by checking a mandatory agreement box before proceeding to the questions.

# S8. Additional robustness and bias diagnostics

For transparency, the supplementary material also records the main additional diagnostics reported in the revised manuscript: Harman single-factor variance explained = 31.8%; full-collinearity VIF range = 1.82–2.64; latent method factor comparison showed only modest fit improvement (ΔCFI = 0.005; ΔRMSEA = -0.005; Δβ = 0.02–0.05). These results suggest that common method bias could not be fully excluded, but was unlikely to materially distort the main structural findings.

Alternative model and robustness checks included comparison of the main serial mediation model with a parallel mediation model, reversed-order mediation model, and no-direct-path model; substitution of selected subjective indicators with archival proxies; alternative estimation with MLR and WLSMV; subgroup analyses in frontline and management samples; and a trimmed-sample analysis excluding the upper and lower 5% of observations.

# S9. Recommended linkage to the main manuscript

- Methods, Section 2.5: add “The full bilingual disclosure version of the focal questionnaire items is provided in Supplementary Appendix Table S1.”
- Methods, Section 2.5.5: add “Control-variable coding is summarized in Supplementary Appendix Table S3.”
- Methods, Section 2.3 / Results, Section 3.1: add “The sample screening flow is summarized in Supplementary Appendix Section S6.”
- Methods, Section 2.9: add “Ethics and data-governance details are summarized in Supplementary Appendix Section S7.”
- Results, Section 3.6: add “Additional robustness and bias diagnostics are summarized in Supplementary Appendix Section S8.”
